# Tablet-Camera Iris Landmarks and EEG/PPG-Derived Arousal Indices During Naturalistic Reading: Geometric Confounding and Exploratory Within-Subject Associations

**DOI:** 10.3390/s26206505

**Published:** 2026-10-14

**Authors:** Sujin Ko, Jaepyo Park, Mincheol Whang, Dongkook Kim, Moonsook Kim

**Affiliations:** 1 Department of Financial Technology Convergence, Soongsil University, Seoul 06978, Republic of Korea; gloriatic57@gmail.com; 2 HiStranger, Inc., Seoul 04147, Republic of Korea; dk@hi-str.com (D.K.); luna@hi-str.com (M.K.)

**Keywords:** non-contact oculometry, iris landmarks, tablet camera, MediaPipe, EEG band power, PPG heart-rate variability, arousal estimation, naturalistic reading, ground-truth sensitivity, geometric confounding

## Abstract

Arousal, the activation dimension of the valence–arousal circumplex, indexes alertness and engagement independently of pleasantness. We evaluated whether MediaPipe FaceLandmarker iris landmark distances from a tablet camera estimate electroencephalography (EEG)- and photoplethysmography (PPG)-derived arousal markers during naturalistic reading. Recordings from 48 participants yielded 43,923 thirty-second windows. Raw iris distances correlated with EEG alpha (r=+0.118) and theta (r=+0.076), but face scale explained 93% of their variance. After face-width normalisation, alpha remained associated (r=+0.076), whereas theta was not statistically significant. Further adjustment for ocular luminance and head pose rendered alpha nonsignificant (r=+0.021, p=0.33). Circular-shift testing identified alpha coupling in 11/48 participants under full control, versus 28/48 using uncorrected Pearson tests. Within-subject Ridge regression, with no raw sample shared between the training and test windows, yielded a nonsignificant group mean correlation (r=+0.078, p=0.07); 13/48 participants had individually significant prediction–reference associations (11 positive), fewer when the PPG term was restricted to filter-consistent beats. Both counts are exploratory, nominal and uncorrected across participants, and come from different analyses. Neither establishes prospective monitoring performance, and the study does not validate the landmark distance as a pupil measure.

## 1. Introduction

Affective computing systems that monitor emotional state in real time have applications in human–computer interaction, mental health assessment, driver monitoring and educational technology [1,2]. Affective states are conventionally placed on the valence–arousal circumplex [3]: valence denotes pleasantness, *arousal* the degree of physiological activation. The two are largely independent, and this study takes arousal as the primary construct because it is the dimension most directly indexed by autonomic and cortical measures.

The dominant paradigm relies on contact sensors—electroencephalography (EEG), photoplethysmography (PPG), electrodermal activity and infrared eye tracking—which give reliable signals but impose electrode preparation, movement artefact and discomfort over long sessions and which cannot be distributed at scale. A method running on a device already in the user’s hands would remove that barrier, and pupil size is the obvious candidate: it is a well-established correlate of cortical arousal [4,5], covarying with EEG band power [6], with the amplitude of evoked cortical responses [7], with autonomic activation [8] and with cognitive load [9]. Google’s MediaPipe FaceLandmarker [10] exposes 478 facial landmarks, including a dedicated iris group at video frame rates, and has accordingly been treated as putting non-contact pupillometry within reach of any consumer camera. We therefore first assessed whether the landmarks measure pupil diameter before evaluating their physiological associations.

### 1.1. Related Work

Pupil–cortex coupling is well characterised in controlled settings. Hong et al. [7] found that pre-stimulus pupil diameter correlates with the trial-to-trial variability of post-stimulus EEG components at 175–200 and 350–400 ms in an auditory oddball task and that pre-stimulus alpha power relates negatively to the evoked pupil dilation. Urai et al. [5] studied pupil-linked arousal in perceptual decision-making, relating pupil responses to choice behaviour and decision bias; van Kempen et al. [11] examined behavioural and neural signatures modulated by the same arousal signal. Podvalny et al. [6] characterised the spectral signature of spontaneous pupil-linked arousal shifts, reporting that beta and gamma power rise with pupil size, while alpha and theta follow an inverted-U, and that cortical activity leads fast pupil events by about 400 ms rather than following them. The physiological basis is the link between pupil size and neuromodulatory activity: pupil fluctuations track adrenergic and cholinergic activity in cortex [12], and general treatments of pupillometry [13] and reviews of eye-tracking metrics in emotional and cognitive processes [14] survey the measures involved. Recent work has extended pupil-linked arousal beyond the psychophysics laboratory—to cortical activity and cognition in Alzheimer’s disease [15], to interactive conversation [16], to ratings of musical groove [17] and to effortful speech listening [18]—and multimodal affective computing has combined ocular with autonomic channels [19] and pursued per-individual model adaptation [20]. These studies use laboratory infrared pupillometry or dedicated eye trackers; whether a consumer camera can stand in for them is a separate question.

Camera-based physiological sensing has advanced on two fronts. Remote photoplethysmography recovers heart rate and heart-rate variability (HRV) from facial colour change [21,22,23], and camera-based sympathetic arousal estimation has been validated against electrodermal and PPG references [24]. Separately, webcam pupil measurement has become an active area: PupilSense estimates pupil diameter from ordinary webcam video, locating the eye region with MediaPipe and predicting diameter with a learned model, evaluated in 51 participants against pupil diameter recorded by an eye tracker [25]. Automated facial action unit (AU) analysis has been applied to continuous valence–arousal prediction [1,2,26], including landmark-based recognition under partial occlusion [27], and hybrid brain–computer interfaces have combined ocular and cortical signals [28,29].

What has not been performed, to our knowledge, is to evaluate the MediaPipe iris landmarks themselves as an ocular measure against concurrently recorded physiology at this sample size in a fully naturalistic setting—including what the landmarks do and do not resolve, which Section 3.2 shows is the decisive question for this measurand.

### 1.2. Engineering Question, Research Questions and Hypotheses

The engineering question is not whether pupil dynamics carry physiological information, which is established for laboratory pupillometry, but whether a MediaPipe-derived ocular measure from a tablet camera retains enough of it, under naturalistic conditions, to serve as a deployment-time substitute for contact sensors. Answering it requires both modalities recorded simultaneously: the camera signal is the candidate sensor under test, and the contact EEG and PPG sensors provide the reference against which it is compared. The reference is a set of biosignal-derived indices, not a measurement of experienced affect (Section 2.5).

We evaluate the camera signal on three descriptive criteria, stated here during revision rather than registered in advance: whether a group-level association is detectable; how many individual subjects show within-subject coupling significant at a nominal level; and whether a per-subject model tracks the reference on held-out data. The second is a count, not a test of prevalence: this study does not test whether the proportion of affected subjects exceeds what the error rate would produce.

Three research questions follow. RQ1: does a MediaPipe-extracted iris-landmark measure exhibit reproducible coupling with EEG band power and PPG indices, at group and individual level, in a naturalistic reading task? RQ2: does the observed coupling depend on the specific choice of biosignal-derived arousal composite? RQ3: can a per-subject regression from camera features to the arousal reference track it on held-out data, and how much does accuracy vary across individuals?

The corresponding hypotheses, also formulated during revision, are as follows: H1, the camera-derived iris diameter correlates significantly with EEG band power and PPG indices at the group level; H2, the coupling between the ocular measure and an arousal composite depends on how that composite is defined; H3, within-subject regression models achieve significant arousal tracking in a subset of subjects, with accuracy varying across individuals rather than uniformly.

## 2. Materials and Methods

### 2.1. Participants and Setting

The dataset comprises recordings from 48 participants, originally collected at HiStranger, Inc. as part of internal product development and quality improvement activities (Project 10031).

**De-identification and what it applies to.** The term is used throughout and describes one specific object, so we separate three stages.

*The source archive.* Recordings are held by HiStranger, Inc. in access-restricted storage, and each session is keyed there by the account e-mail address used to register for the application. This material is identifiable and remains so; nothing in this study de-identifies it.

*Feature extraction.* Deriving the features analysed here required reading those recordings. The original extraction produced the landmark and biosignal features, and the two re-processing passes of Section 3.2 and Section 3.3 read the archived video again offline to derive the face-scale, luminance and intensity-ratio quantities the archived feature set did not contain. The retained outputs of every pass are numerical: no frame, crop or still image was kept, and no participant image appears in this article. During processing, the e-mail key is resolved to a sequential pseudonym (S01–S48) in memory; that mapping is not written to disk, and every file, table and figure produced by this study are keyed by the pseudonym alone.

*The analytical dataset.* What the statistical analyses in Section 3 and Section 4 operate on is a de-identified, feature-level analytical dataset: per-window ocular and facial features, image-intensity summaries, EEG band-power and PPG interval indices and the age and sex of Table 1, indexed by pseudonym. It contains no images and no direct identifiers.

We write “de-identified” rather than “anonymous” deliberately. The description applies to the analytical dataset, not to the source recordings, and because those recordings and their e-mail keys continue to exist under the data controller’s access restriction, re-identification is prevented by that access control rather than by the irreversibility of the data themselves.

**Participant flow.** Forty-nine participants took part. For one, no facial video was recorded, so that participant is absent from every analysis, giving N=48. The exclusion is a missing-modality event fixed at acquisition: in the primary analyses no signal-quality, coupling-direction or tracking-performance criterion was applied. A PPG signal-quality criterion enters only the sensitivity analysis of Section 3.5, which reports the subjects it retains. Separately, 1 of the 48 was initially absent from the two re-processing passes of Section 3.2 and Section 3.3 because the verification step compared the re-extracted and archived series at zero time offset only; that recording begins 450 s into the session, and once the offset is estimated, the two series agree at r=+0.9997. All 48 are included in every primary analysis. We state the criterion explicitly because a zero-lag comparison alone would have rejected a recording that is in fact the correct one.

**Task and schedule.** Participants read scenario-based material on a tablet in an office setting, without task instructions beyond reading. Recording was organised as two group sessions rather than individual appointments: five participants on 25 November 2025 and 43 on 27 November 2025, recorded concurrently in the same room with start times spanning 14:25–14:40. Time of day is therefore near-constant across subjects. That is not the same as holding circadian phase constant, since internal phase at a given clock time varies with chronotype and habitual sleep timing, neither of which was recorded (Section 4.3).

**What was and was not controlled.** Participant characteristics are given in Table 1. Because the recordings originated as product-development sessions, the record is limited to those variables. Participants were asked in advance to abstain from caffeine and nicotine, to have had adequate sleep and not to be taking psychotropic medication; these instructions were verbal, and compliance was not verified. Iris pigmentation, refractive correction and pre-existing ocular conditions were neither instructed nor recorded. The age range (19–46) excludes older adults, in whom senile miosis and reduced pupillary dynamic range would be expected.

### 2.2. Apparatus and Acquisition

**Figure 1:**
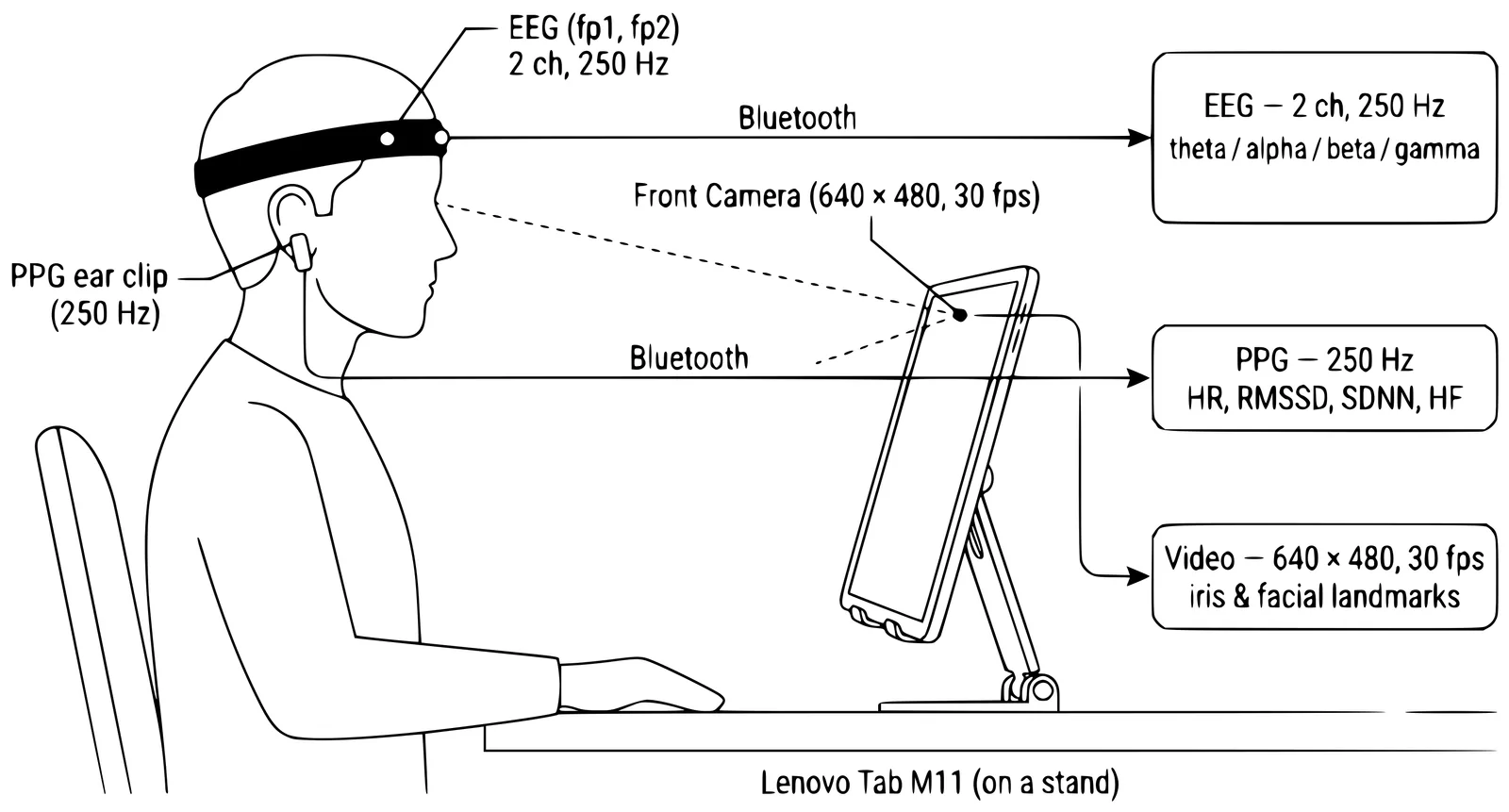
Recording setup (schematic; no participant is depicted). The seated participant read on a Lenovo Tab M11 held in portrait orientation on an adjustable desk stand at a nominal viewing distance of about 40 cm. A consumer headband carried two prefrontal electroencephalography (EEG) electrodes (fp1, fp2) and an ear-clip photoplethysmography (PPG) sensor, both streamed over Bluetooth to the tablet at 250 Hz, while the tablet’s built-in front camera recorded the face at 640×480 and 30 fps; the dashed lines indicate the camera’s view of the face. The contact sensors were instrumented only to provide the physiological reference against which the camera signal is evaluated—the proposed pipeline uses the camera alone at deployment. The display did not move during a session, but the participant was unrestrained: no chin rest or headrest was used and no fixed viewing distance was enforced, so the face-to-camera distance varied with posture (Section 3.2). Participants were recorded concurrently in a shared room rather than individually (Section 2.1).

**Camera.** Participants read on a Lenovo Tab M11 (11.0-inch, 1920×1200) held in portrait orientation on an adjustable desk stand at a nominal 40 cm viewing distance with no head restraint. The 8 MP f/2.0 front module recorded 640×480 video at approximately 30 fps. Figure 1 shows the arrangement.

**EEG and PPG.** Two-channel prefrontal EEGs (fp1, fp2) and a single-channel PPG were recorded at a nominal 250 Hz from a consumer wearable headband. Two products were used: 27 participants on a unit supplied by SOSO H&C and 21 on an OMNIFIT Brain unit (Omni C&S). The units supplied through both sources were identified by the authors as the neuroNicle FX2; the OMNI C&S user manual for that model names LAXTHA as the contract manufacturer and lists Korean medical-device licence 16-4837 [30]. That identification does not establish identical hardware revisions, firmware or measured frequency responses across the units, and the recordings show they are not the same device: the two transmit in different packet formats and quantise the PPG channel at different bit depths, so decoding the archive required a separate parser for each, and the preprocessing sources branch on the product: the OMNI path normalises each EEG channel over the whole recording and detrends the PPG with a one-second moving average before filtering, neither of which the SOSO path does. Channel-wise normalisation can change the relative contributions of the two channels to averaged band power, and its effect on the reported associations was not quantified. For one of the 48 recordings, the stored EEG is consistent with that normalisation step; for the rest, the branch that executed is not recorded, so we do not claim that the two products received identical preprocessing. Supplementary Section S1 gives the packet formats, the two preprocessing paths with what we could and could not verify about them, and the product comparison. Assignment to products was not randomised; it followed which units were free, so product is confounded with participant.

The device records mono-polar with reference and ground on a clip at the right earlobe; the PPG sensor is built into that same ear clip and operates in transmission mode, so EEG and PPG are co-sampled from one unit. Frontal electrodes are dry metal contacts requiring no gel. The manufacturer specifies a 3–41 Hz passband at −3 dB, an input range of ±590 μV at 0.036 μV resolution, 4 ms inter-beat-interval precision and a 30–200 beats min^−1^ pulse range. The SOSO frame also carries three 16-bit accelerometer words per sample, which the OMNI frame does not. Their vector magnitude is nearly constant within each of the 27 SOSO recordings, at a level consistent with gravity, which we take as confirming the decoding (Supplementary Section S1).

**Preprocessing.** EEG was band-pass filtered 3–50 Hz with cascaded fifth-order Butterworth stages applied with zero-phase forward–backward filtering, followed by outlier removal (>4 SD) and cubic spline interpolation of missing samples. No independent component analysis (ICA) was applied: with a two-channel prefrontal montage, the number of recorded channels does not exceed the number of concurrent artefact sources, so an ICA decomposition is not identifiable in this configuration. Ocular and movement artefacts were instead addressed by the high-pass stage, amplitude-based rejection and exclusion of windows with more than 10% missing samples. The device passband is narrower than the software filter: the upper end of the gamma band defined below (40 Hz) lies below the published −3 dB corner but close to it, and we did not measure the response of the units used here, so the attenuation inside that band is unquantified. Gamma is used in no primary analysis.

PPG was band-pass filtered 0.3–1.2 Hz with the same cascaded fifth-order zero-phase construction. Beat detection used NeuroKit2 [31], whose default pipeline applies a further 0.5–8 Hz stage, so the passband reaching the peak detector is the intersection, 0.5–1.2 Hz, or 30–72 beats min^−1^. The cohort median inter-beat interval is 815 ms (73.6 beats min^−1^), placing the cardiac fundamental for a typical participant at the upper edge of that passband. This restriction originates in our preprocessing, not the instrument. Section 3.5 reports what it costs the interval-derived features.

**PPG signal quality.** A band-pass filter does not establish that the filtered signal carries a usable pulse: motion artefact that is large relative to the pulsatile component passes the filter and displaces the detected beats. At revision we therefore scored every analysis window for residual artefact. Scoring used the archived raw stream filtered 0.5–8 Hz rather than the analysis band, because the analysis band reduces the pulse to a near-sinusoid and so hides the waveform distortion that artefact produces. For each 30 s window we computed three quantities: the mean correlation of each pulse with the window’s average pulse (template matching [32]); three physiological feasibility rules from the same source (heart rate 40–180 beats min^−1^, longest inter-beat interval below 3 s, ratio of longest to shortest interval below 2.2); and the fraction of samples in runs of identical value lasting 0.1 s or longer, which marks saturation or dropout. A window passes if its template correlation is at least 0.86 [32], it meets all three rules, and fewer than 5% of its samples are flat. The threshold is the published one and was not tuned on these data. As continuous descriptors we also recorded waveform skewness [33], the share of raw-signal power between 1 and 2.25 Hz, and the agreement of two beat detectors [34]; all indices were computed with NeuroKit2 [31].

A clean waveform in the 0.5–8 Hz band does not by itself make the stored intervals correct, because those were detected in the analysis band. We therefore also report, per window, the agreement between the beats detected in the two bands (F1 score, ±150 ms tolerance). The analysis-band beats are re-detected from the raw stream, as in the re-derivation of Section 3.5. The product-dependent preprocessing is not re-executed, so these are not the stored beats themselves. For the 27 SOSO recordings, the standard deviation of accelerometer magnitude within a window gives a measure of head movement that does not depend on the PPG. Windows are those of the stored features: in all 48 recordings the raw stream has the same length as the stored one, and the product identified from the frame format matches the recorded device. Quality screening was added after the analyses were fixed and enters only the sensitivity analysis of Section 3.5; no window was excluded from the primary analyses.

**Timing.** Both biosignal streams reach the tablet over Bluetooth rather than a hardware-clocked acquisition, so the realised rate carries packet-level jitter around a median of 249 samples s^−1^. Stream onsets are aligned to the video using recorded timestamps. Downstream windowing and feature extraction then operate on sample index at the nominal 250 Hz: windows are formed as ⌊30×FS⌋ samples with start times start/FS, and the nominal rate is passed to the spectral and beat-detection routines. We did not resample to a common clock or correct for drift within a recording, and we have not quantified the residual timing error; it is listed as a limitation.

### 2.3. Software

Processing used Python 3.14 with MediaPipe 0.10, OpenCV 4.13, SciPy 1.17, NeuroKit2 0.2.13, scikit-learn 1.8, NumPy 2.4 and pandas 2.3. The bivariate Granger test was implemented directly rather than taken from a library: a vector autoregression at lag three fitted by ordinary least squares (numpy.linalg.lstsq), with restricted and unrestricted residual sums of squares compared by an *F*-test (scipy.stats). No separate PC was involved in recording; both biosignal streams were transmitted over Bluetooth directly to the recording tablet. Offline processing ran on an Apple M4 Pro with 64 GB RAM under macOS 26.5.

### 2.4. Camera Features

Ocular and facial features were extracted per frame with MediaPipe FaceLandmarker (face_landmarker.task, 478-point mesh). The primary ocular measure is the iris diameter (ID), the Euclidean distance between horizontal iris landmark endpoints—469 and 471 for the left eye, 474 and 476 for the right—in normalised image coordinates.

**Figure 2:**
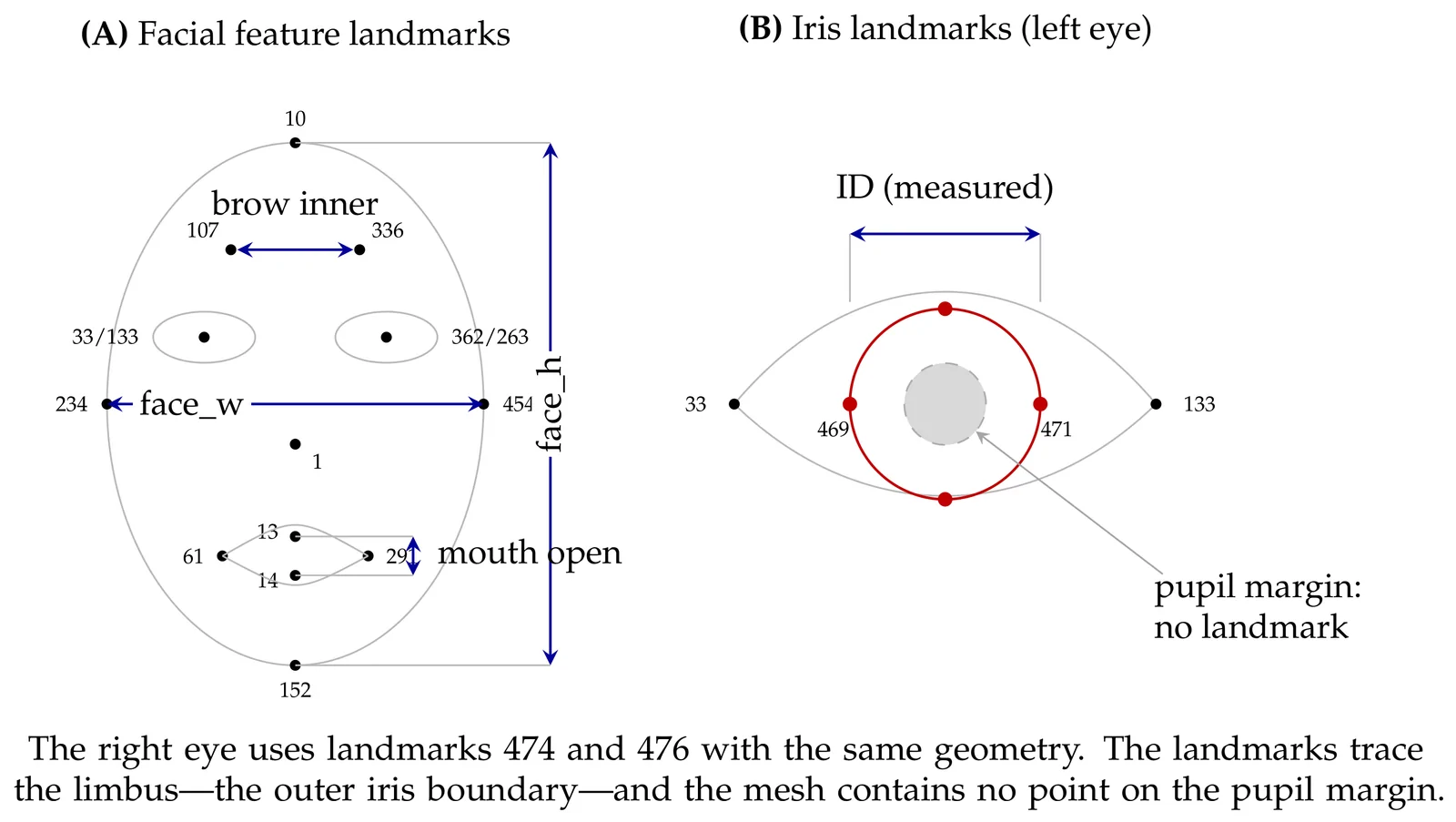
Landmark positions used for feature extraction (schematic; no participant image is reproduced). (**A**) The MediaPipe indices from which the facial and head-pose features and the face-scale references of Section 3.2 are computed. (**B**) The iris landmarks at the centre of Section 2.4: 469 and 471 sit on opposite sides of the limbus, the outer iris boundary, and the distance between them is the measure this study reports as the iris diameter (ID). The pupil margin, drawn dashed, has no corresponding landmark in the mesh and is not localised by the model.

We name it explicitly because the distinction governs the interpretation of everything that follows. The MediaPipe iris sub-model returns the iris (limbus) boundary, not the pupil margin [35]; the library’s documentation uses the resulting diameter as a metric scale reference precisely because the horizontal iris diameter is approximately constant across a wide population, which it gives as 11.7±0.5 mm [36]. What this feature captures is therefore not pupil size. At the 640×480 capture resolution, the iris spans 21.2±2.7 image pixels (median 21.1, range 13.7–29.1), so a 0.5 mm change in pupil diameter corresponds to about 0.9 pixels. We did not validate the pupil-boundary accuracy of any extractor at this resolution, and we make no general claim about what sub-pixel estimation can achieve; we report that our measure does not attempt it. Figure 2 shows the landmarks used and where the pupil margin lies relative to them.

Blinks were detected from the vertical eyelid separation normalised by face height, with a frame counted as a blink when that ratio falls below 0.015. This is not the eye aspect ratio (vertical over horizontal distance), which enters the model separately as a feature and is itself an established landmark-based ocular index [37]. Blink frames were linearly interpolated and smoothed with a kernel-5 median filter.

Nine further features were extracted from the same mesh: eye aspect ratio, inner-brow distance (AU4 proxy), left and right brow raise (AU1/2), mouth-corner height (AU12/15), mouth openness (AU25/26) and head yaw, pitch and roll. The first six are geometric approximations of the movement associated with the corresponding action unit; the three head-pose angles index gross orientation rather than any action unit. Landmark geometry is not trained Facial Action Coding System (FACS) coding, and all nine are normalised by face width or height. We make no claim that they agree with trained or appearance-based AU intensity coding, which we did not test; Supplementary Section S4 gives the landmark definitions. Together with the four ocular features (ID_mean, ID_std, ID_slope, ID_entropy), these give the 13 camera-derived features used throughout.

### 2.5. Biosignal Features and Reference Construction

EEG band power was computed with Welch’s method over 30-s sliding windows at a 5-s step, in four bands per channel: theta (4–8 Hz), alpha (8–13 Hz), beta (13–30 Hz) and gamma (30–40 Hz), with fp1/fp2 averages formed per window. Windows with more than 10% missing samples were excluded. Frontal alpha asymmetry (FAA) was computed as $\ln\left(\mathrm{fp}2_{\alpha }\right)-\ln\left(\mathrm{fp}1_{\alpha }\right)$, following the standard formulation [38,39]. PPG features over matching windows were the root mean square of successive RR-interval differences (RMSSD), the standard deviation of NN intervals (SDNN), the mean NN interval (MeanNN) and high-frequency (HF) band power; the low-frequency (LF)/HF ratio was excluded because the window is too short for reliable LF estimation.

**What the reference is and is not.** No subjective assessment was collected: no self-report, no experimenter rating, no independent psychological annotation. What we measure are physiological indices computed from EEG and PPG. The construct-validity chain has two links, and this study tests one: *camera features → physiological indices*. The link we do not test is *physiological indices → subjective experience*, for which we rely on prior validation of each constituent [8,40,41,42,43,44]. For readability, we write “arousal” and “valence” for the operational indices throughout; wherever those words appear without qualification, they denote these indices and not measured affective experience. A statement such as “13 of 48 subjects showed a significant arousal association” means that, for those subjects, the camera-based prediction correlated with the biosignal-derived index beyond the circular-shift null, in either direction; we call an association *tracking* only when it is positive.

The reference indices are

(1) $$\mathrm{arousal}=\frac{1}{2}\left[z(\mathrm{avg}\_\mathrm{beta})-z(\mathrm{RMSSD})\right],\;\mathrm{valence}=\mathrm{FAA},$$

with z(·) denoting within-subject *z*-scoring over the session. RMSSD is the time-domain index most influenced by vagally mediated variability, so it falls as vagal tone withdraws [44], and frontal beta power enters as an index of cortical activation: beta power has been reported to rise with pupil-linked arousal [6], although the functional account of the beta band is not a simple activation one [43]. Beta responses have also been reported to differ in direction between participant groups defined by a trait measure [45]. The sign of the beta term is therefore a modelling convention of this composite rather than an established mapping, which is one reason we report sensitivity to the composite’s definition. The two modalities are recorded by separate instruments from the camera, which removes shared *measurement* apparatus; it does not establish that their errors are statistically independent of the camera features, and Section 4.3 returns to a path by which they may not be. Because the choice of composite is one of several defensible formulations, we evaluate sensitivity to it across seven alternatives (Supplementary Section S2).

### 2.6. Statistical Analysis

Per-subject Pearson *r* values were computed between ID_mean and each biosignal feature. Fisher *z*-transformation was applied before group-level inference, with $H_{0}$: mean r=0 tested by a one-sample *t*-test over one value per subject. The reported group intervals are $\tanh(\bar{z}\pm 1.96\;s_{z}/\sqrt{n})$, a normal approximation on the Fisher-*z* scale rather than a *t*-based interval, so an interval and the *t*-test *p*-value beside it can disagree marginally at n=48.

Rows with a missing value in any variable a given analysis needs are dropped before that analysis, so the retained rows are not always contiguous in time. Therefore, 10 of the 48 subjects have at least one gap wider than the nominal 5 s, reaching 75 s at the widest. Every test operates on the order of the retained rows, so the serial structure it assumes is an approximation of the recording’s time structure, and the circular-shift test of Section 2.7 rotates by rows rather than by seconds.

**Within-subject prediction.** A Ridge regression model (α=1.0) was fitted for each participant using the first 80% of that participant’s time-ordered windows and evaluated on the last 20%, with the 13 camera features as predictors. Feature standardisation used statistics calculated from the training windows only. A window labelled *t* covers the raw interval [t,t+30) s, and windows begin every 5 s, so a chronological split without purging would leave the last five training windows drawing on raw signal that also enters the test period. We discard those five: the last retained training window ends exactly at the first test window’s start. Verified on timestamps across all 48 subjects, the interval between the last training sample and the first test sample is 0.0 s and never negative. The training set contains a median of 709 windows and the test set 179 (range 110–262).

Two sources of residual dependence remain. The reference composites are *z*-scored over the whole session, which involves the test period. Rescaling the constructed composite on training rows changes nothing, because a correlation is invariant to affine transformation of either series (verified numerically; maximum difference $2\times 10^{-15}$). Normalising *each component* on training statistics before differencing is not an affine transformation of the composite: it changes the relative weight of the two terms and so defines a different target, which we report separately in Section 3.4. Moreover, zero-phase filtering is applied over the whole recording, so test-period signal influences training-period values; the purge leaves a zero gap and provides no margin against it. Widening the gap by discarding further training windows moves the group mean between +0.073 and +0.087 out to 300 s of separation and to +0.062 at 600 s, with the nominal *p* crossing 0.05 in both directions while the training set shrinks; participant-level performance rankings remained highly correlated with those in the primary analysis throughout (Spearman ρ≥0.96 out to 300 s). We interpret this as instability of a small effect under a shrinking sample rather than as evidence that the residual dependency is or is not material, since gap and training size are not separated. Supplementary Section S2.3 gives the six conditions.

**Cross-subject prediction.** Leave-one-subject-out cross-validation was run for Ridge, support vector regression (SVR) and random forest. Successive windows from one participant share that participant’s anatomy, camera geometry and baseline, so a randomly partitioned *k*-fold split over windows would place the same participant in both folds and let the model recognise the individual rather than the state; a subject-grouped *k*-fold would avoid that as well, and we do not claim leave-one-subject-out is the only valid scheme. One condition must be stated: input features are *z*-scored within participant over each participant’s *whole* session before the split, so the held-out participant’s own full-session statistics—including the period being predicted—enter their features. The leave-one-subject-out figures are therefore retrospective and are not an estimate of performance on a new user whose future data are unavailable.

### 2.7. The Circular-Shift Test

Per-subject significance is assessed against a circular-shift null rather than an ordinary Pearson *p*-value. The test was added during revision, after the original analysis had been run and its results seen; it is a correction to the inferential procedure, not a pre-registered choice.

**Why.** Windows are 30 s long and advance in 5 s steps, so neighbours share 25 s of their data, and successive values of any window-level series are strongly dependent. A Pearson *p*-value assumes independent draws and is anticonservative under that dependence.

**The null and its limits.** For two standardised window-level series *x* and *y* of length *n* with $\hat{\rho }=\frac{1}{n}\sum_{t}x_{t}y_{t}$, a null replicate rotates one against the other, $x_{t}^{\left(s\right)}=x_{(t-s)\;\mathrm{mod}\;n}$. The rotation preserves the *circular* autocorrelation of *x* exactly at every lag. It preserves the ordinary autocorrelation only to the extent that the series behaves as though its end continued into its beginning, which a 30–100 min reading session satisfies only approximately: a slow session-long drift creates a discontinuity at the wrap that the unshifted series does not contain. The null is therefore approximate.

**Enumeration.** We enumerate the admissible shift set rather than sample it. All shifts with 12≤s≤n−12 are used, which is n−23 values: 87 to 239 per subject for the held-out series and 523 to 1283 for full-session series. Excluding |s|<12 windows (60 s, twice the window length) keeps the observed alignment out of the null. Enumerating the set makes the computation deterministic; it does not make the test exact with respect to the null, which remains the approximate one described above. Correlations for all shifts are computed together as a circular cross-correlation through the fast Fourier transform (FFT).

**The *p*-value.** Two-sided, with *K* the number of enumerated shifts, $p=\left(1+\#\{s:\right|{\hat{\rho }}_{s}\left|\geq \right|\hat{\rho }\left|\}\right)/\left(1+K\right)$, counting the observed statistic in both numerator and denominator [46]. Its lower bound is 1/(1+K), so no *p*-value is reported as zero or below that bound.

**Covariate control and multiplicity.** Where a coupling is reported under control, both series are first residualised on the covariates within subject and the shifting is applied to the residuals; the covariates are not shifted. No correction is applied across subjects or bands, and per-subject counts are reported as descriptive results at a nominal 5% level. We do not compare them against the number a 5% error rate would produce: that is a group-level statement about how many people in the population are affected, and a count of participants meeting the nominal significance threshold does not supply one.

**An alternative null.** We also ran a Fourier phase-randomised surrogate null, which retains the amplitude spectrum of the prediction and randomises its phases (5000 surrogates, seed 0). This preserves the periodic autocovariance and keeps *n* fixed; it does not remove the periodic-boundary assumption, and it is a different approximate null rather than an independent check on correctness [47]. We report its counts alongside the circular ones wherever an individual-level claim is made. A third variant, which truncates the wrapped tail instead of rotating it, returns far lower counts (3 of 48 for arousal). Its replicates are correlations over series of different lengths, and the larger sampling variance of the short ones can widen the reference distribution; we did not establish the false-positive rate of that variant, so we neither adopt it nor read its low counts as support for the circular test. Supplementary Section S2.1 reports it in full.

### 2.8. Use of Generative Artificial Intelligence

The authors directed the analyses and reviewed the results. Anthropic Claude was used to assist with English-language editing, code verification and reanalysis. The authors reviewed the AI-assisted work and take full responsibility for the manuscript.

## 3. Results

### 3.1. Data Overview

Preprocessing and feature extraction yielded 43,923 valid 30-s windows across 48 subjects (mean 915±203 windows per subject; range 549–1310). MediaPipe returned a landmark set in 99.9% of frames on average (SD 0.3%; lowest subject 97.9%), and the mean non-blink valid-pupil ratio was 97.0% (SD 1.7%; range 91.6–99.6%). These figures establish that the capture side of a tablet-only pipeline works under office lighting; they say nothing about the accuracy or physiological validity of what was captured.

### 3.2. Group-Level Association and Its Geometric Decomposition

Table 2 gives the group-level correlations between ID_mean and each biosignal index for the measure as submitted. EEG alpha (r=+0.119, p=0.002) and theta (+0.077, p=0.018) reach significance; beta, RMSSD and HF do not. Moreover, 31 of 48 per-subject correlations are positive for each of alpha and theta.

The measure is a landmark distance in image coordinates, so it scales with the apparent size of the face and therefore with viewing distance. Because no head restraint was used, that quantity varied freely within and between sessions. We reprocessed the source recordings to extract per-frame face width, face height, inter-ocular distance and mean grey level of the face and eye regions, none of which the archived feature set contained.

**How much of the measure is geometry.** Within subjects, the iris diameter tracks face width almost perfectly: mean r=+0.964 (median +0.977, range +0.728 to +0.997), exceeding 0.7 in 48 of 48 subjects, mean $R^{2}=0.932$. The within-subject coefficient of variation falls from 0.130 for the raw measure to 0.029 after division by face width. Roughly nine tenths of the variance in the submitted measure is face scale, which, at fixed anatomy, is camera distance.

**What survives control.** Table 3 repeats the group-level correlation under successively stricter control. The alpha association survives face-width normalisation at about two thirds of its magnitude; theta does not. Normalising instead by inter-ocular distance leaves alpha short of significance, and adding ocular luminance and head pose removes it as well. We therefore withdraw the group-level claim rather than restate it in weaker terms.

**Individual level.** Against the circular-shift null, alpha coupling is significant in 8 of 48 subjects for the raw measure, 7 of 48 after face-width normalisation and 11 of 48 under full control; the phase-randomised null returns 10 of 48 under full control. Restricting the analysis to every sixth window, so that no two analysed windows share a raw sample, gives 6 of 48 when the minimum rotation is held at the same 60 s used throughout—this removes the window overlap but not the autocorrelation of the underlying signals, so it is corroboration rather than a clean control. Among the 11, the coupling is bidirectional (6 positive, 5 negative, |r| from 0.15 to 0.35), which is why it cancels under group averaging. These are descriptive counts at a nominal level.

### 3.3. An Exploratory Alternative Measurand

An intensity-ratio measure requiring no pupil boundary—the ratio of mean grey level over a central disc of the iris to that over a surrounding annulus—was examined as an exploratory alternative measurand. After controlling movement, eyelid aperture, luminance and head pose, it retains small negative associations with alpha (−0.052) and theta (−0.046) at nominal p<0.05, with 13 of 48 subjects individually significant under the circular-shift test. No reference pupil diameter was available, so it has not been compared against a ground-truth measurement, and we make no claim about its accuracy; several bands and control levels were examined, and the *p*-values are nominal. Supplementary Section S3 gives the method and the full table.

### 3.4. Within-Subject and Cross-Subject Prediction

*What enters the model.* Thirteen camera-derived features per 30-s window: four ocular features and the nine facial action-unit proxies listed in Section 2.4. No EEG or PPG value is available to the model.

*What it predicts.* The biosignal-derived arousal or valence index of Equation (1) for the same window.

*Training and evaluation.* One model per subject, fitted on the first 80% of that subject’s windows and evaluated on the last 20%, with the five boundary windows purged (Section 2.6). Accuracy is the Pearson correlation between predicted and reference trajectories over the held-out portion.

*Prediction performance.* The prediction–reference correlation for arousal had a group mean of r=+0.078, which was not statistically significant (p=0.069); 13 of 48 subjects reach individual significance under the circular-shift null and 10 of 48 under the phase-randomised null, with 10 significant under both. Of the 13, 11 track in the positive direction (median r=+0.312, maximum +0.668) and 2 in the negative. Section 3.5 examines how far this count depends on the PPG term and on PPG signal quality. Valence gives a group mean of +0.084 (p=0.037) with 11 of 48 and 10 of 48 under the two nulls (Table 4).

**Figure 3:**
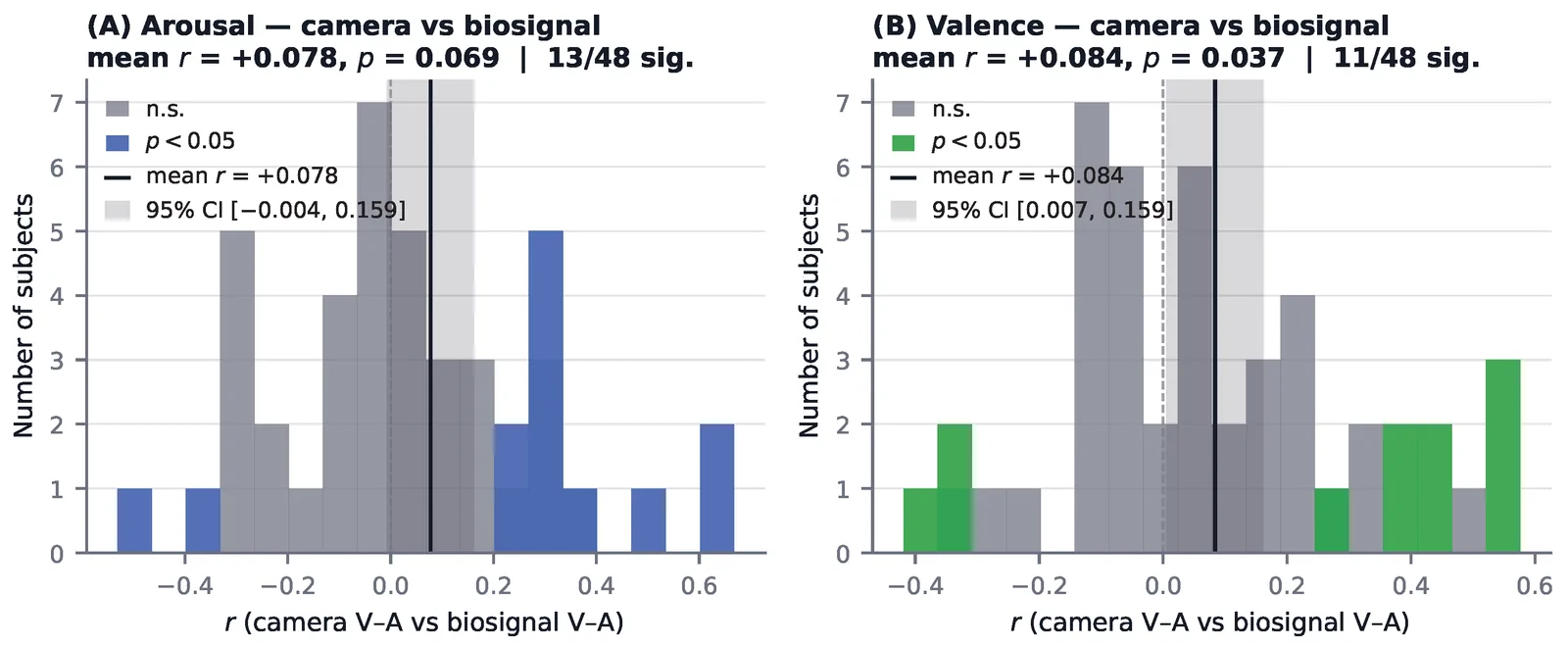
Distribution of per-subject *r* between the camera-predicted and biosignal-derived indices (within-subject Ridge regression, chronological 80/20 split with the five-window purge of Section 2.6). Coloured: significant under the circular-shift test at a nominal p<0.05 (13/48 for arousal, 11/48 for valence); gray and “n.s.”: not significant. Shaded band: 95% confidence interval of the group mean *r*. The counts are descriptive and are not corrected for testing 48 subjects.

Figure 3 shows the distribution of per-subject accuracies and their relationship to session length. The distributions are wide and centred only slightly above zero, which is what produces the modest group mean. Accuracy and the number of windows a subject contributed are not significantly correlated, which is a failure to detect a relationship rather than evidence that session length is unrelated to accuracy.

*Cross-subject prediction.* Under leave-one-subject-out cross-validation, arousal retains a positive group mean (Ridge r=+0.107±0.140; SVR +0.085±0.115; random forest +0.074±0.121; mean ± SD over the 48 held-out subjects), whereas valence sits at approximately zero for every model (Ridge −0.059±0.178; SVR +0.009±0.093; random forest +0.003±0.081). One condition qualifies these figures: input features are *z*-scored within participant over each participant’s whole session before the split, so the held-out participant’s own full-session statistics enter their features. They are retrospective estimates, not estimates of performance on a user whose future data are unavailable.

### 3.5. Reliability of the Reference and Robustness Checks

**The interval-derived PPG features.** Because the acquisition passband is narrow relative to the cardiac waveform, we re-derived the HRV features from the archived raw streams for all 48 participants, under the same 0.3–1.2 Hz band and again under a wider 0.5–8 Hz band. The re-derivation is not a re-run of the original pipeline: it detects beats with ppg_process, discards intervals outside 300–2000 ms and computes RMSSD directly, where the stored values came from hrv_time without that interval filter, and the product-dependent preprocessing of Section 2.2 was not re-executed. It orders subjects almost identically to the stored values (Spearman ρ=0.96 for RMSSD) but does not reproduce their magnitudes, so the three sets are reported separately rather than treated as one quantity.

Summarising each subject by the median over that subject’s windows, the stored RMSSD values have a cohort median of 131 ms and fall in the 20–100 ms range expected of a resting adult for 20 of 48 subjects. The narrow-band re-derivation gives a median of 182 ms and 15 of 48 and exceeds a quarter of that subject’s own mean interval in 21 of 48; its MeanNN is 824 ms and lies in the 600–1200 ms range for 46 of 48 subjects. The wide-band re-derivation gives a median of 41 ms and 35 of 48. The stored values are the ones the primary composite is built on; the re-derivations are diagnostics of the passband, not replacements.

We report this as a failure to establish a reliable HRV reference, not as a diagnosis. No electrocardiogram was recorded, so neither band’s intervals have been checked against a reference, and a value falling inside a population range is not evidence that it is correct. The two bands do not order subjects the same way: Spearman ρ=−0.31 between the stored values and the wide-band re-derivation and −0.23 between the narrow- and wide-band re-derivations. We therefore treat both as unvalidated and do not substitute one for the other. RMSSD is half of the primary arousal composite, and SDNN and HF depend on the same interval series.

**Signal quality and motion artefact.** Of the 43,923 windows, 89.5% passed quality screening (per subject: median 98.5%, range 0–100%), and four subjects had fewer than half of their windows pass. In two recordings no window passed. In one, the PPG channel stayed at the upper limit of its range throughout, so no pulse was recorded. The EEG from the same unit was within the cohort range, which points to saturation of the optical channel, although a device fault cannot be excluded. The stored PPG-derived features of that recording are therefore not physiological, and it accounts for 1,310 of the 1,340 windows that failed the flat-signal criterion. In the other, a pulse component was present but too weak for consistent beat detection. Of all failing windows, 86.9% failed a feasibility rule and 47.9% the template criterion; a window can fail more than one. Template correlation fell as movement rose: in 26 of the 27 SOSO recordings its within-subject Spearman correlation with accelerometer variability was negative (median ρ=−0.36). Supplementary Figure S1 shows raw, analysis-band and 0.5–8 Hz traces for a passing and a failing window, with the beats each band’s detector found, for one SOSO and one OMNIFIT participant, each the one whose pass fraction is closest to its product’s median. Each window is the one at the median template correlation of its group, not the clearest example.

Screening the waveform and checking the stored intervals are different questions. In the 42,613 windows of the 47 recordings with a pulse, beats detected in the analysis band agreed with those detected in the 0.5–8 Hz band (F1 ≥0.95) in 23,568 (55.3%); median agreement was 0.96. Disagreement tracks the implausibility of the stored values. Within each of the 47 recordings with a pulse, lower agreement went with higher stored RMSSD (Spearman ρ negative in all 47, median −0.76). Across subjects, median agreement was 1.00 for the 20 whose stored RMSSD lies in the 20–100 ms resting range and 0.93 for the 27 outside it. Most windows in which the two bands disagree still pass quality screening (16,119 of 19,045, 84.6%, against 23,177 of 23,568, 98.3%, of windows in which they agree), so the disagreement is not explained by detectable artefact alone. It is a disagreement between two re-detected beat series, neither validated against an electrocardiogram, and it does not show which series is in error. What it shows is that intervals derived from these recordings depend on the passband, and that this dependence goes with the implausibility of the stored values.

**Does the within-subject result depend on the RMSSD term?** We repeated the analysis of Section 3.4 against a reference containing no PPG—z(beta) alone—keeping the model, split and test unchanged, and again with the head-pose features removed and with the ocular features alone, since beta is the band most exposed to movement-linked myogenic activity. The count of individually significant subjects is 16, 14 and 14 of 48 across the three variants (15, 14 and 13 positive), against 13 and 11 for the primary reference, and the group mean rises from +0.078 to +0.248, +0.223 and +0.174. The result therefore does not rest on the RMSSD term, on the facial features or on the head-pose predictors. It does not follow that what remains is free of movement or of viewing geometry: the four ocular features are the submitted ones in raw image coordinates, and removing head-pose *predictors* does not remove the geometric variation inside ID_mean itself. We keep the pre-defined composite as the primary reference and report these as sensitivity checks.

**Sensitivity to signal quality.** We repeated the within-subject analysis of Section 3.4 and the two PPG rows of Table 2 under three conditions: windows with any PPG signal (no flat run), screened windows, and screened windows on which the two bands also agree (F1 ≥0.95). The model, features, split and test were unchanged. Failing windows were masked rather than removed, so that the circular-shift test (Section 2.7) keeps the time structure it relies on: the reference is rotated together with its mask, and the correlation is taken over the windows still valid after rotation. With no window masked this reproduces the primary results exactly. The split point is that of the primary analysis. The model is fitted on valid training windows only, and the composite is re-standardised over the retained windows. A subject is excluded from a condition when fewer than 60 valid training or 40 valid test windows remain (60 valid windows for the correlations). Because exclusion falls on the subjects with the poorest signals, each condition is compared with the all-window result for the same subjects (Table 5).

Excluding the recording without a pulse leaves every row essentially unchanged; that its group *p* falls below 0.05 for arousal prediction is not a change we interpret. Screening lowers the count of individually significant subjects for arousal prediction from 13 to 10 of 46. Masking costs power whatever the masked windows contain, so we compared each screened result against 500 masks of the same size and run structure, obtained by rotating each subject’s actual mask to a random position within the training and the test portion. The rotations are seeded by replicate and condition, so the null is reproducible; with 500 replicates the smallest attainable *P* is 1/501≈0.002. All random-mask *P* values are one-sided, the proportion of rotated masks giving a result at or below the observed one. For quality-screened windows the observed count lies inside that distribution (median 12, 95% range 9–14; P=0.17 of 10 or fewer), and so does the group mean (+0.073 against a median of +0.086). The reduction is therefore compatible with the loss of windows alone. This control can show only that it did not detect a contribution from motion-contaminated windows, not that they made none. Requiring the two beat series to agree as well gives results that depend on the endpoint and the threshold. At an agreement threshold of 0.95, five of 34 subjects are significant against a random-mask median of eight (P=0.042), four with a positive coefficient against six (P=0.21), and the group mean is +0.067 against +0.087 (P=0.28). At 0.90, seven of 42 are significant against ten (P=0.10), three with a positive coefficient against seven (P=0.002), and the group mean is +0.030 against +0.092 (P=0.012). Three of these six uncorrected comparisons have P<0.05. Windows in which the two beat series disagree may therefore have contributed to prediction of the RMSSD-containing composite, but the evidence does not establish it. This is consistent with the conclusion above that the interval-derived features are not a reliable reference. The analysis against z(beta) alone, which uses no PPG, is unaffected. The correlations of ID_mean with RMSSD and HF show no group-level association under any condition.

**Sensitivity to the composite.** Across seven alternative arousal composites, the group-level ocular–composite coupling changes sign with the definition: the vigilance and broadband-shift indices and the cardiac-interval index give negative group means; the inverted theta–alpha ratio a positive one. Mean |r| lies between 0.12 and 0.22. A group coefficient computed against a composite therefore cannot be reported without naming the composite. The raw ocular–band correlations are unchanged under every definition, because no composite enters their computation; that is an arithmetic identity and not evidence of robustness. Supplementary Section S2 gives the full table.

**Other analyses.** Facial features showed small group-level associations with the arousal reference at nominal significance (eye openness +0.070, head pitch +0.065, head roll −0.044), examined across nine features without correction for multiplicity; a bivariate Granger analysis found temporal precedence of ID_mean over EEG theta and alpha in a subset of subjects. Both are exploratory and reported in Supplementary Section S4. The Granger *F*-tests are computed over the same overlapping windows corrected elsewhere here, their residual and stationarity assumptions were not checked, and the 30 s window at a 5 s step cannot resolve the sub-second lead reported for pupil-linked cortical effects; we therefore do not use them as evidence of physiological precedence.

## 4. Discussion

### 4.1. What the Camera Recovers and What It Does Not

The engineering question was whether a tablet front-facing camera with open-source MediaPipe iris landmarks can recover physiologically meaningful arousal-related signals in naturalistic conditions. Two factors limit what these data support: the geometric sensitivity of the measurand and the serial dependence between analysis windows.

The first is the measurand. The raw landmark distance does correlate with EEG alpha and theta at the group level, but it tracks face width at r=+0.964 within subjects, so most of what it varies with is the participant’s distance from the camera. Normalising by face width reduces the alpha correlation to +0.076, while the theta association is no longer statistically significant; adding ocular luminance and head pose reduces alpha to +0.021, which was also not statistically significant. We do not claim a group-level ocular–cortical coupling.

The second is the inference. Per-subject significance on 30-s windows advancing in 5-s steps cannot be read from a Pearson *p*-value. Against a circular-shift null, 11 of 48 subjects retain alpha coupling under full geometric control, and 13 of 48 show a significant within-subject association with the arousal index (11 positive, 2 negative); a phase-randomised null gives 10 of 48 for each. These are descriptive counts at a nominal level. We do not claim the effect is present in more people than chance allows—that is a group-level statement, and we do not test it—and neither count is a majority.

What this study contributes can be stated in three sentences. Face scale explained most of the variance in the raw iris landmark distance. The group-level alpha association was not statistically significant after adjustment for face width, ocular luminance and head pose, although 11 of 48 participants met the nominal circular-shift significance threshold. Separately—a different analysis, on the purged split rather than under geometric control—the within-subject association with the arousal index met that threshold in 13 of 48 participants, 11 positive and 2 negative, at accuracies around |r|=0.3 in those subjects. Part of that count may rest on windows in which the beats detected in two filter bands disagree, while against an EEG-only reference 16 of 48 met the threshold (Section 3.5). These exploratory findings do not establish prospective monitoring performance.

We do not extend any of the three to the validity of the measure as pupillometry or to accuracy in use. The remainder of this discussion proceeds from that result. It concerns a physiological index rather than experienced emotion, fatigue or attention, since no report of what participants felt was collected (Section 2.5).

**The hypotheses.** H1 predicted a significant group-level correlation, and on the measure as submitted, that correlation is present. It does not survive geometric control, so the raw correlation was observed while an association independent of viewing geometry was not supported. H2 predicted that ocular–composite coupling depends on how the composite is defined, and it does. For H3, we report the findings descriptively without classifying it as supported or unsupported: accuracy varies across individuals, widely and in both directions, and the prediction–reference association survives the circular-shift test in 13 of 48 subjects (11 positive). We do not determine whether these findings support H3, since no proportion was specified in advance, and applying one now would be a threshold chosen after seeing the result.

**Why the per-subject correlations differ.** The per-subject values differ in sign—most clearly for beta, 28 positive against 20 negative—so a signed group mean over correlations of both signs is smaller than the typical absolute correlation. Opposite-signed correlations reduce the signed group mean without implying a physiological mechanism, and their origin was not established: estimation noise alone produces correlations of both signs, dispersion does not pull the expected value of a sample mean toward zero, and a small directional mean is equally consistent with weak coupling in most subjects as with opposing couplings of some strength. Restricted variation in underlying state, measurement noise in landmarks recorded in image coordinates, and heterogeneity of the coupling itself are all candidates; we did not quantify any of their contributions.

### 4.2. Comparison with Prior Work and Practical Limits

**What the group-level numbers do not support.** The group figure of r=+0.119 is a mean of 48 per-subject correlations, not a correlation over pooled data, so it does not convert into a proportion of variance accounted for, and we report no such quantity. The mean within-subject correlation was about 0.12 before geometric adjustment and was not statistically significant after it, so it supports no inference about an individual user, and the practical utility of a single population-level model was not established here. The same applies to the cross-subject results, where the best model reaches r=+0.107 under a retrospective normalisation condition.

**What the individual-level numbers support.** Among the 11 subjects whose positive within-subject tracking survives the circular-shift test, median accuracy is r=+0.312, and the best subject reaches +0.668. These subjects were identified against the composite reference, whose PPG term is not a reliable reference (Section 3.5). Accuracy is reported as a Pearson correlation; a prediction-error based $R^{2}$ was not evaluated, and the squared correlation between a prediction and a reference is not that quantity. The subgroup was defined by inspecting the same held-out accuracies that summarise it, so it describes this cohort under post hoc selection and is not a validated pass rate for any screening procedure. Establishing one would require selecting users on one session and evaluating them on another, which this dataset does not support.

The distinction that matters for applications is between a per-window state readout and a session-level trend, and this study addresses neither. A correlation does not by itself establish per-window prediction accuracy or monitoring utility: that would require the prediction errors, a stated decision rule and a criterion for acceptable performance, none of which we specify or evaluate. We therefore make no claim about whether a per-window readout at these accuracies could support a statement about a user at a given moment and none about whether a session-scale trend is recoverable; the latter would require a study built around slow trends with an independent measure of them.

Table 6 places this study against camera-based ocular and affective work. The entries do not share an outcome metric, a reference standard or a task, and we make no numerical comparison across rows; the columns of interest are the reference standard and the affective range the task affords. We are the only entry measuring the iris landmark distance directly rather than using it to locate the eye region for a separate diameter model, as PupilSense does [25], and Section 3.2 quantifies the consequence of that choice.

**Deployment.** EEG and PPG were recorded as reference signals only; the proposed pipeline needs only the tablet camera. The near-ceiling landmark-detection and valid-frame rates establish that *capture* is feasible under unconstrained office lighting. They establish nothing about monitoring utility. We did not evaluate fatigue or attention monitoring, we did not fit or test a prospective calibration procedure, and we did not compare a per-user model against a population one on independent data. Whether per-user fitting confers an advantage that generalises across sessions is therefore an open question. The per-subject models reported here were fitted and evaluated within a single session; cross-session performance was not assessed.

A calibration-gated design—fitting a per-user mapping at enrolment, measuring its held-out accuracy and monitoring only users whose accuracy exceeds a threshold—is the approach these results motivate, and Supplementary Section S5 sets out what evaluating it would involve. We describe it as an untested candidate design. No screening rule was specified in advance or tested on independent data here.

**Computational cost.** On a desktop machine (Apple M4 Pro, CPU inference), end-to-end throughput for the complete per-frame path—decode, MediaPipe inference with iris refinement and all thirteen features—averaged 146 frames s^−1^ (median 145, range 100–179 across 31 sessions), or 6.8 ms per frame. The landmark model is 3.6 MB. This is a desktop benchmark; we did not measure throughput on the tablet, and the desktop margin does not establish that on-device real-time operation is achievable.

### 4.3. Limitations and What Would Resolve Them

**No subjective measure.** The references are physiological indices; no self-report, experimenter rating or independent annotation was obtained. In some subjects the camera-based prediction was associated with an EEG- and PPG-derived index (13 of 48 nominally significant, 11 of them positive), and the PPG term of that index is not established as a reliable reference (Section 3.5); the study does not establish that the camera recovers the index in general. Nor does it establish that the camera recovers emotion, and whether those indices track experienced arousal in this population and task is assumed from prior validation of their constituents rather than tested here. A reading task engages cognitive load and oculomotor demands that modulate frontal beta and pupil size without a corresponding change in felt affect. The conservative reading of this work is as a sensor-agreement study rather than an affect-recognition study.

**The reference itself.** The interval-derived PPG features are not established as reliable at the level of an individual subject (Section 3.5), and RMSSD is half of the primary arousal composite. A replication should record a reference electrocardiogram, or widen the acquisition passband, before deriving interval statistics. The EEG side rests on a two-channel consumer montage, adequate for prefrontal band power but not for source-level claims. Moreover, while the reference is recorded by separate instruments from the camera, that does not make its errors statistically independent of the camera features: head and body movement degrades beat detection and also moves the landmarks, so a movement-driven component could appear in both. For the SOSO recordings an accelerometer now measures part of that path: PPG quality falls with head movement (Section 3.5). Whether the same movement drives the camera features was not tested, and for the OMNI recordings the path is unmeasured. We therefore still do not argue that reference noise makes our counts conservative. The quality screen was not validated against an electrocardiogram, and template matching cannot reject artefact that is itself periodic at a plausible heart rate.

**Design and sample.** All recordings come from two group sessions on two afternoons in one room. Time of day is near-constant, but internal circadian phase at a given clock time varies with chronotype and sleep timing, neither of which was recorded; circadian differences are therefore among the explanations we cannot exclude rather than among those ruled out. There is no test–retest measurement, so the stability of a subject’s coupling across occasions is unknown. Environmental conditions are confounded with the study as a whole. The reading task elicits a narrower band of affective states than evocative paradigms, which may have affected the associations, though we did not quantify that contribution. The 48 participants were a convenience sample of working adults with no inclusion or exclusion criteria applied, so the figures were obtained with the confounds present rather than removed; that makes them the more relevant kind of number for a consumer–hardware pipeline, but it does not convert a post hoc proportion into a prospective one.

**Measurement and analysis.** The luminance control is image-based: mean grey level of the eye region responds to exposure and gain as well as to scene illuminance and is not a measurement of light reaching the retina, so residual light-reflex variance may remain. Windowing and feature extraction operate on sample index at the nominal 250 Hz rather than on a resampled common clock, and the residual timing error is unquantified. The 30-s window precludes analysis of rapid phasic transients; whether 30 fps suffices for such work would need its own validation, and we attempted none. The circular-shift null is approximate, and the phase-randomised alternative is a second approximate null rather than an independent check; the two differ in the counts they produce, and we report both. Per-subject counts throughout are descriptive at a nominal level, computed without correction for multiplicity across subjects, bands or composites. The intensity-ratio, facial-feature and Granger analyses are exploratory and were selected from a larger set of examined comparisons.

**Why some subjects show no coupling.** The complementary groups—37 subjects without alpha coupling under full control, 35 without a significant arousal association—are the majority. We compared the two groups on each of the three variables we recorded, separately for each of the two splits, and detected no significant difference in any of the six comparisons: session length, age and sex. Age did not predict coupling strength in either split. Supplementary Table S11 gives the six comparisons with the split each belongs to, since the two splits contain different subjects and their statistics are not interchangeable. Failing to detect a difference in 11 or 13 subjects against 35 or 37 is not evidence that the groups do not differ.

One numerical difference is worth recording, though it is not significant: 10 of the 11 subjects retaining alpha coupling are female, against 32 of 48 in the cohort (Fisher’s exact p=0.07 for that split; p=1.00 for the arousal split). It is one of eight uncorrected comparisons, and eleven subjects could not support an inference about sex differences even had it reached significance. The explanations we cannot test—fatigue, iris pigmentation, refractive correction, medication, chronotype—were not recorded, and distinguishing them requires a prospective protocol that measures them.

## 5. Conclusions

We evaluated a tablet-camera, MediaPipe-based non-contact oculometry pipeline against simultaneously recorded EEG and PPG references in a 48-subject naturalistic reading task (43,923 thirty-second windows). Three findings carry forward. First, the ocular quantity this pipeline extracts is the inter-landmark iris diameter, not pupil diameter, and most of its variance is viewing geometry: within subjects it tracks face width at r=+0.964. Second, once that geometry is removed, the group-level association does not survive: the theta association is no longer statistically significant after face-width normalisation, and alpha falls from r=+0.118 to +0.021 (p=0.33) under full control. Third, what remains is individual and modest—11 of 48 subjects retain alpha coupling under full control, and 13 of 48 show a significant within-subject association with the arousal index against a circular-shift null (11 positive, 2 negative), 10 of 48 against a phase-randomised one, with a median accuracy of +0.312 among the 11 positive.

The contribution is that account, made quantitative on 48 participants. The two individual-level counts above come from different analyses—geometric control for the alpha coupling, the purged split for the arousal association—and neither is evidence that the measure is pupillometry, nor an accuracy a deployed system should expect.

Two measurement cautions follow for anyone extending this line of work. A landmark-based ocular measure reported in image coordinates should be reported after explicit geometric normalisation, because most of its variance is not ocular. Furthermore, individual-level significance computed on overlapping windows should be tested against the serial structure of those windows. We also report that the interval-derived PPG features of this dataset are not established as a reliable reference, which bears on any composite built from them: restricting the arousal composite to windows whose beats agree across filter bands lowered the arousal-prediction result by more than the loss of windows explains for some endpoints and thresholds but not for others (Section 3.5). After quality screening 10 of 46 subjects were individually significant (13 of the same 46 on all windows), and 5 of 34 when the two bands were also required to agree (11 of the same 34).

We regard the individual-level residual and the intensity-ratio measure as exploratory. Neither has been validated against an independent pupil measurement, which is the step this design cannot supply. Whether per-user calibration recovers more than a population model, and whether any of this tracks experienced affect, are open questions requiring prospective studies designed around them.

## Figures and Tables

**Table 1:** Participant characteristics (N=48).

Variable	Value
Age	30.6±6.8 years (range 19–46)
Sex	32 female, 16 male
Session length	median 77 min (range 46–110)
Occupation	office workers, freelancers, students
Not recorded	iris pigmentation, refractive correction, ocular conditions,
	chronotype, verified caffeine/nicotine/sleep/medication status

**Table 2:** Group-level ID_mean ↔ biosignal correlations (N=48), Fisher-*z* one-sample test over one *r* per subject.

Signal	Mean *r*	*p*	Sig. Subjects	
			Pearson	Circular
EEG alpha	+0.119	**0.002**	37/48	8/48
EEG theta	+0.077	**0.018**	33/48	5/48
EEG beta	+0.019	0.556	29/48	4/48
PPG RMSSD	−0.030	0.198	29/48	6/48
PPG HF	+0.023	0.086	25/48	3/48

Group columns are unaffected by window overlap, being computed over one *r* per subject. The Pearson counts treat overlapping windows as independent draws and are inflated; the circular counts are descriptive at a nominal 5% level (Section 2.7).

**Table 3:** Group-level correlation of the ocular measure with EEG band power under successive geometric controls (N=48; Fisher-*z* one-sample test).

Control	EEG Alpha		EEG Theta	
	Mean r	p	Mean r	p
None (as submitted)	+0.118	0.002	+0.076	0.019
÷face width	+0.076	0.017	+0.044	0.170
÷inter-ocular distance	+0.034	0.171	+0.008	0.743
÷face width + luminance	+0.061	0.039	+0.030	0.297
÷face width + luminance + head pose	+0.021	0.326	−0.002	0.929

Every row is computed on the windows for which all geometric covariates are defined, so the rows are comparable to one another; the small third-decimal differences from Table 2, which uses each subject’s full window set, are the same quantities on slightly different samples.

**Table 4:** Within-subject prediction of the biosignal reference from camera features (N=48) and the same analysis under two constructions of the reference composite.

	Arousal	Valence
Mean *r* (Fisher *z*)	+0.078	+0.084
95% confidence interval (CI)	[−0.004,0.159]	[0.007,0.159]
*p* (group level)	0.069	0.037
*Subjects individually significant, nominal p<0.05*		
Pearson (inflated by window overlap)	28/48	24/48
Circular-shift	**13/48**	11/48
Phase-randomised surrogate	10/48	10/48
*Arousal reference constructed two ways*		
Normalised over the session (as defined; retrospective)	+0.078 (p=0.069), 13/48, 11 positive	
Components normalised on training rows (forward)	+0.130 (p=0.010), 14/48, 12 positive	

The last two rows are different targets, not a corrected and uncorrected version of one quantity, so the difference between them does not measure a bias; 45 of 48 subjects receive the same significance verdict under both. Neither is a fully independent prospective validation, since zero-phase filtering crosses the split in both (Section 2.6).

**Table 5:** PPG-dependent results under quality screening. Group mean *r* is the Fisher-*z* average of one *r* per subject, tested against zero. “Same subjects” repeats the all-window analysis on the subjects retained by the row above it. Sig. counts subjects with circular-shift p<0.05.

Analysis	Windows	*N*	Mean *r* [95% CI]	*p*	Sig.
Arousal prediction	all	48	+0.078 [−0.004, +0.159]	0.069	13
	signal present	47	+0.087 [+0.004, +0.169]	0.046	13
	same subjects, all	47	+0.081 [−0.003, +0.163]	0.064	13
	screened	46	+0.073 [−0.013, +0.159]	0.104	10
	same subjects, all	46	+0.085 [0.000, +0.169]	0.057	13
	screened, bands agree	34	+0.067 [−0.031, +0.164]	0.188	5
	same subjects, all	34	+0.122 [+0.020, +0.222]	0.025	11
ID_mean–RMSSD	all	48	−0.030 [−0.074, +0.015]	0.198	6
	signal present	47	−0.031 [−0.076, +0.015]	0.196	6
	same subjects, all	47	−0.030 [−0.075, +0.016]	0.208	6
	screened	46	−0.029 [−0.077, +0.018]	0.233	5
	same subjects, all	46	−0.029 [−0.075, +0.017]	0.228	6
	screened, bands agree	41	−0.011 [−0.054, +0.033]	0.627	3
	same subjects, all	41	−0.025 [−0.074, +0.024]	0.318	6
ID_mean–HF	all	48	+0.023 [−0.003, +0.049]	0.086	3
	signal present	47	+0.024 [−0.003, +0.051]	0.084	3
	same subjects, all	47	+0.024 [−0.003, +0.051]	0.085	3
	screened	46	+0.023 [−0.005, +0.051]	0.118	4
	same subjects, all	46	+0.025 [−0.002, +0.052]	0.078	3
	screened, bands agree	41	+0.030 [−0.004, +0.063]	0.088	4
	same subjects, all	41	+0.028 [−0.001, +0.056]	0.065	3

**Table 6:** This study in relation to camera-based ocular and affective work. The entries do not share an outcome metric, a reference standard or a task, so the reported outcomes are not mutually convertible and no numerical comparison across rows is intended; the columns of interest are the reference standard and the affective range that the task affords.

Study	Ocular/Camera Measure	Reference Standard	Task	*N*	Reported Outcome
Jiang et al. 2022 [28]	Pupillary response, infrared eye tracker + EEG	Cued target (known ground truth)	12-target low-frequency VEP paradigm	10	94.90±2.34% accuracy; 64.35±3.07 bits/min (supervised, 1.5 s)
Yi et al. 2024 [29]	Pupil constriction and gaze, eye tracker + P300	Instructed response	Clinical communication, disorders of consciousness	10 healthy, 10 patients	100% accuracy in the healthy group; 76.1±7.9% in the five patients who could use it
Odinaev et al. 2023 [23]	Facial video (vascular, not ocular)	Contact PPG	Public remote PPG (rPPG) datasets	—	Mean absolute error 10.5 ms for RMSSD and 6.15 ms for SDNN
Shah et al. 2024 [25]	Pupil diameter: MediaPipe locates the eye region, a learned regression model estimates diameter from the RGB crop	Pupil diameter from a Tobii eye tracker at 90 Hz	Webcam sessions	51	Mean absolute percentage error 3.2–3.6% under leave-one-participant-out
Nomiya et al. 2025 [26]	Facial action units (17 AUs, appearance-based, OpenFace)	Self-reported valence and arousal ratings	Deliberate expression of remembered states (training); film clips (test)	48 train, 23 test	Predictions correlate positively with the ratings in both sets (p<0.001); the per-subject *r* values are reported graphically
**This study**	Iris-landmark diameter, tablet camera at 640×480, no infrared (IR)	EEG- and PPG-derived indices (no subjective report)	Uninstructed naturalistic reading, 77 min	48	Group alpha r=+0.118 raw, +0.076 after face-scale normalisation and not statistically significant under full control; 11/48 subjects individually significant; 13/48 significant arousal associations, 11 of them positive, median r=+0.312

## Data Availability

The de-identified, feature-level analytical dataset on which the reported statistics were computed—per-window ocular and facial features, image-intensity summaries, EEG and PPG indices and the demographic variables of Table 1, indexed by pseudonymous participant codes—is available from the corresponding author on reasonable request and execution of a standard data-use agreement. The source facial recordings are identifiable material held by HiStranger, Inc. and cannot be shared. The analysis scripts that produce the reported statistics, including the significance test of Section 2.7 and the split verification of Section 2.6, are available on request.

## Supplementary Materials

The following supporting information can be downloaded at: https://www.mdpi.com/article/10.3390/s26206505/s1, Table S1: Comparison of the two headband products; Table S2: Within-subject arousal prediction as the gap between the training and test portions widens; Table S3: The within-subject analysis under two constructions of the reference composite; Table S4: Alternative arousal ground-truth definitions evaluated in the sensitivity analysis; Table S5: Pupil (ID_mean) and alternative arousal ground-truth definitions; Table S6: Section 3.4 of the main article repeated against an arousal reference containing no PPG; Table S7: PPG signal quality and arousal prediction per subject; Figure S1: Raw, analysis-band and 0.5–8 Hz PPG for one passing and one failing window from each product; Table S8: Group-level correlation between the intensity ratio C and EEG band power under cumulative control; Table S9: Facial features extracted as action unit (AU) proxies; Table S10: Facial features and EEG/PPG-derived arousal and valence at the group level; Figure S2: Group-level correlations between the nine non-ocular features and the biosignal-derived arousal and valence indices; Figure S3: Biosignal-derived and camera-predicted arousal and valence for one subject; Figure S4: Proportion of subjects whose VAR(3) *F*-test for precedence of ID_mean over each biosignal reaches nominal p<0.05; Figure S5: The raw measure, before geometric normalisation; Figure S6: Distribution of per-subject correlations between ID_mean and each biosignal target, before geometric normalisation; Figure S7: Per-subject held-out accuracy for the arousal index; Table S11: Subjects reaching nominal significance compared with those not reaching it, for each of the two splits; Figure S8: Leave-one-subject-out accuracy for three model families; Figure S9: Proposed three-stage personalised calibration pipeline.

## Abbreviations

The following abbreviations are used in this manuscript:

AU	Action unit
BCI	Brain–computer interface
EAR	Eye aspect ratio
EDA	Electrodermal activity
EEG	Electroencephalography
ERS	Event-related synchronisation
FAA	Frontal alpha asymmetry
FACS	Facial action coding system
HF	High frequency
HRV	Heart-rate variability
ICA	Independent component analysis
ID	Iris diameter (inter-landmark)
LC-NE	Locus coeruleus–norepinephrine
LOSO	Leave-one-subject-out
MeanNN	Mean NN interval (inverse of heart rate)
PPG	Photoplethysmography
RMSSD	Root mean square of successive differences
rPPG	Remote photoplethysmography
SAM	Self-Assessment Manikin
SDNN	Standard deviation of NN intervals
SVR	Support vector regression
VAR	Vector autoregression
V–A	Valence–arousal

## References

[1] Soleymani M., Asghari-Esfeden S., Fu Y., Pantic M. (2016). Analysis of EEG signals and facial expressions for continuous emotion detection. IEEE Trans. Affect. Comput..

[2] Saffaryazdi N., Wasim S.T., Dileep K., Nia A.F., Nanayakkara S., Broadbent E., Billinghurst M. (2022). Using facial micro-expressions in combination with EEG and physiological signals for emotion recognition. Front. Psychol..

[3] Russell J.A. (1980). A circumplex model of affect. J. Personal. Soc. Psychol..

[4] Joshi S., Li Y., Kalwani R.M., Gold J.I. (2016). Relationships between pupil diameter and neuronal activity in the locus coeruleus, colliculi, and cingulate cortex. Neuron.

[5] Urai A.E., Braun A., Donner T.H. (2017). Pupil-linked arousal is driven by decision uncertainty and alters serial choice bias. Nat. Commun..

[6] Podvalny E., King L.E., He B.J. (2021). Spectral signature and behavioral consequence of spontaneous shifts of pupil-linked arousal in human. eLife.

[7] Hong L., Walz J.M., Sajda P. (2014). Your eyes give you away: Prestimulus changes in pupil diameter correlate with poststimulus task-related EEG dynamics. PLoS ONE.

[8] Bradley M.M., Miccoli L., Escrig M.A., Lang P.J. (2008). The pupil as a measure of emotional arousal and autonomic activation. Psychophysiology.

[9] van der Wel P., van Steenbergen H. (2018). Pupil dilation as an index of effort in cognitive control tasks: A review. Psychon. Bull. Rev..

[10] Lugaresi C., Tang J., Nash H., McClanahan C., Uboweja E., Hays M., Zhang F., Chang C.L., Yong M.G., Lee J. (2019). MediaPipe: A framework for building perception pipelines. arXiv.

[11] van Kempen J., Loughnane G.M., Newman D.P., Kelly S.P., Thiele A., O’Connell R.G., Bellgrove M.A. (2019). Behavioural and neural signatures of perceptual decision-making are modulated by pupil-linked arousal. eLife.

[12] Reimer J., McGinley M.J., Liu Y., Rodenkirch C., Wang Q., McCormick D.A., Tolias A.S. (2016). Pupil fluctuations track rapid changes in adrenergic and cholinergic activity in cortex. Nat. Commun..

[13] Mathôt S. (2018). Pupillometry: Psychology, Physiology, and Function. J. Cogn..

[14] Skaramagkas V., Giannakakis G., Ktistakis E., Manousos D., Karatzanis I., Tachos N.S., Tripoliti E., Marias K., Fotiadis D.I., Tsiknakis M. (2023). Review of eye tracking metrics involved in emotional and cognitive processes. IEEE Rev. Biomed. Eng..

[15] David M.C.B., Mallas E.J., Li L.M., Kolanko M.A., Nilforooshan R., Tsoi M.L., Karakoc H., Hoang K., Brandt J., Triantafyllou C. (2025). Pupil-linked arousal, cortical activity, and cognition in Alzheimer’s disease. Brain Commun..

[16] Masters B., Aliakbaryhosseinabadi S., Wendt D., MacDonald E.N. (2025). Pupil Responses During Interactive Conversation. Trends Hear..

[17] Spiech C., Sioros G., Endestad T., Danielsen A., Laeng B. (2022). Pupil drift rate indexes groove ratings. Sci. Rep..

[18] Cui M.E., Herrmann B. (2023). Eye Movements Decrease during Effortful Speech Listening. J. Neurosci..

[19] Zhang T., El Ali A., Wang C., Zhu X., Cesar P. CorrFeat: Correlation-based feature extraction algorithm using skin conductance and pupil diameter for emotion recognition. Proceedings of the 2019 International Conference on Multimodal Interaction (ICMI).

[20] Gutiérrez-Martín L., López-Ongil C., Lanza-Gutiérrez J.M., Miranda Calero J.A. (2024). Personalized Clustering for Emotion Recognition Improvement. Sensors.

[21] Yu Z., Li X., Zhao G. Remote photoplethysmograph signal measurement from facial videos using spatio-temporal networks. Proceedings of the British Machine Vision Conference (BMVC).

[22] Poh M.Z., McDuff D.J., Picard R.W. (2010). Non-contact, automated cardiac pulse measurements using video imaging and blind source separation. Opt. Express.

[23] Odinaev I., Wong K.L., Chin J.W., Goyal R., Chan T.T., So R.H.Y. (2023). Robust Heart Rate Variability Measurement from Facial Videos. Bioengineering.

[24] Braun B., McDuff D., Baltrusaitis T., Holz C. (2023). Video-based sympathetic arousal assessment via peripheral blood flow estimation. Biomed. Opt. Express.

[25] Shah V., Watanabe K., Moser B.B., Dengel A. (2024). PupilSense: A Novel Application for Webcam-Based Pupil Diameter Estimation. arXiv.

[26] Nomiya H., Shimokawa K., Namba S., Osumi M., Sato W. (2025). An Artificial Intelligence Model for Sensing Affective Valence and Arousal from Facial Images. Sensors.

[27] Farkhod A., Abdusalomov A.B., Mukhiddinov M., Cho Y.I. (2022). Development of Real-Time Landmark-Based Emotion Recognition CNN for Masked Faces. Sensors.

[28] Jiang L., Li X., Pei W., Gao X., Wang Y. (2022). A Hybrid Brain–Computer Interface Based on Visual Evoked Potential and Pupillary Response. Front. Hum. Neurosci..

[29] Yi Z., Pan J., Chen Z., Lu D., Cai H., Li J., Xie Q. (2024). A Hybrid BCI Integrating EEG and Eye-Tracking for Assisting Clinical Communication in Patients With Disorders of Consciousness. IEEE Trans. Neural Syst. Rehabil. Eng..

[30] OMNI C&S neuroNicle FX2 User Manual. Document ON-UM-004, Rev. 0, 2024. Contract Manufacturer LAXTHA; Korean Medical-Device Licence 16-4837. https://omnifit.co.kr/pdf/BROCHURE_OMNIFIT_MEDICAL_202508.pdf.

[31] Makowski D., Pham T., Lau Z.J., Brammer J.C., Lespinasse F., Pham H., Scholzel C., Chen S.H.A. (2021). NeuroKit2: A Python toolbox for neurophysiological signal processing. Behav. Res. Methods.

[32] Orphanidou C., Bonnici T., Charlton P., Clifton D., Vallance D., Tarassenko L. (2015). Signal-quality indices for the electrocardiogram and photoplethysmogram: Derivation and applications to wireless monitoring. IEEE J. Biomed. Health Inform..

[33] Elgendi M. (2016). Optimal signal quality index for photoplethysmogram signals. Bioengineering.

[34] Ho S.Y.S., Ding Z., Wong D.C., Kristof F., Brimicombe J., Cowie M.R., Dymond A., Lindén H.C., Lip G.Y.H., Williams K. (2025). Accurate RR-interval extraction from single-lead, telehealth electrocardiogram signals. medRxiv.

[35] Ablavatski A., Vakunov A., Grishchenko I., Raveendran K., Zhdanovich M. (2020). Real-time Pupil Tracking from Monocular Video for Digital Puppetry. arXiv.

[36] Google (2020). MediaPipe Iris: Real-time Iris Tracking and Depth Estimation. MediaPipe Solutions Documentation. https://research.google/blog/mediapipe-iris-real-time-iris-tracking-depth-estimation/.

[37] Dewi C., Chen R.C., Chang C.W., Wu S.H., Jiang X., Yu H. (2022). Eye aspect ratio for real-time drowsiness detection to improve driver safety. Electronics.

[38] Davidson R.J. (1992). Anterior cerebral asymmetry and the nature of emotion. Brain Cogn..

[39] Allen J.J.B., Coan J.A., Nazarian M. (2004). Issues and assumptions on the road from raw signals to metrics of frontal EEG asymmetry in emotion. Biol. Psychol..

[40] Reznik S.J., Allen J.J.B. (2018). Frontal asymmetry as a mediator and moderator of emotion: An updated review. Psychophysiology.

[41] Hagemann D., Naumann E., Thayer J.F., Bartussek D. (2002). Does resting electroencephalograph asymmetry reflect a trait? An application of latent state-trait theory. J. Personal. Soc. Psychol..

[42] Klimesch W. (1999). EEG alpha and theta oscillations reflect cognitive and memory performance: A review and analysis. Brain Res. Rev..

[43] Engel A.K., Fries P. (2010). Beta-band oscillations—Signalling the status quo?. Curr. Opin. Neurobiol..

[44] Shaffer F., Ginsberg J.P. (2017). An overview of heart rate variability metrics and norms. Front. Public Health.

[45] Cooper N.R., Simpson A., Till A., Simmons K., Puzzo I. (2013). Beta event-related desynchronization as an index of individual differences in processing human facial expression: Further investigations of autistic traits in typically developing adults. Front. Hum. Neurosci..

[46] Phipson B., Smyth G.K. (2010). Permutation P-values should never be zero: Calculating exact P-values when permutations are randomly drawn. Stat. Appl. Genet. Mol. Biol..

[47] Schreiber T., Schmitz A. (2000). Surrogate time series. Phys. D Nonlinear Phenom..

