# Supplementary material for: Tablet-Camera Iris Landmarks and EEG/PPG-Derived Arousal Indices During Naturalistic Reading: Geometric Confounding and Exploratory Within-Subject Associations
Source: Sensors (Basel). 2026 Oct 14;26(20):6505. doi: 10.3390/s26206505 (PMC13709060; doi:10.3390/s26206505)
Supplement: Supplementary file 1 [file sensors-26-06505-s001.zip › supplementary.pdf]

Supplementary Material

Tablet-Camera Iris Landmarks and EEG/PPG-Derived Arousal Indices during Naturalistic Reading: Geometric Confounding and Exploratory Within-Subject Associations

Sujin Ko, Jaepyo Park, Mincheol Whang, Dongkook Kim and Moonsook Kim

This document contains the instrumentation detail, implementation detail and secondary analyses referenced from the main article. Section numbers in the form "Section 2.4" refer to the main article; those in the form "Section S2" refer to this document. All quantities were computed by the same scripts that produce the main article's numbers, from the single results file `results/final_numbers.json` and the per-subject CSV outputs it summarises. Counts reported here are descriptive at a nominal 5% level and are not corrected for multiplicity, in this document as in the main article.

Contents

S1 Instrumentation and Alignment **2**
S1.1 The two headband products 2
S1.2 Electrical specification and its consequences 3
S1.3 Product comparison 3
S1.4 Sampling rate, timing and alignment 4
S1.5 PPG signal-quality screening 4

S2 Statistical Implementation and Robustness **5**
S2.1 The circular-shift test 5
S2.2 Train/test separation and the boundary purge 6
S2.3 Boundary-gap sensitivity 7
S2.4 The composite construction 7
S2.5 Non-overlapping windows 8
S2.6 Sensitivity to the arousal composite 8
S2.7 The PPG-free ablation 9
S2.8 Sensitivity to PPG signal quality 10

S3 The Intensity-Ratio Measure **11**

S4 Secondary and Exploratory Analyses **13**
S4.1 Facial features at the group level 13
S4.2 A single-subject illustration 14
S4.3 Cross-lagged and Granger analyses 15
S4.4 Group means and coupling magnitude before geometric normalization 16
S4.5 Subjects with and without significant coupling 17
S4.6 Cross-subject models 18

S5 An Untested Candidate Design for Per-User Calibration **18**

S1 Instrumentation and Alignment

S1.1 The two headband products

Two-channel prefrontal EEG (fp1, fp2) and single-channel PPG were recorded at a nominal 250 Hz from a consumer wearable headband. Two products were present across the cohort: 27 participants were recorded with a unit supplied by SOSO H&C and 21 with an OMNIFIT Brain unit (Omni C&S). The authors identified the units supplied through both sources as the neuroNicle FX2. The OMNI C&S user manual for that model (document ON-UM-004, Rev. 0, 2024) names OMNI C&S as the specification developer, LAXTHA as the contract manufacturer, and Korean medical-device licence 16-4837; the LAXTHA product page lists the same model and number. The two products are therefore the same certified model rather than independently developed instruments, and the electrical specification below is the one published for it.

That is a statement about the model, not about the individual units. A shared model name and licence number do not establish that the hardware revision, the firmware or the measured frequency response was identical across the units we used, and we found no manufacturer document describing the SOSO supply route itself, so we do not describe that distribution relationship from public sources.

We stop short of calling them the same device, because the recordings show that they are not identical. The two transmit in different packet formats—a 33-byte frame delimited by `0xA0`/`0xC0` in the SOSO stream against a 20-byte frame headed by `0xFF 0xFE` in the OMNIFIT stream—and they quantize the photoplethysmographic channel differently, at 24 bits signed and 15 bits unsigned respectively. Decoding the archive required a separate parser for each. The SOSO frame also carries three 16-bit signed words after its eight 24-bit channels (bytes 26–31 of the 33-byte frame). We decode these as a three-axis accelerometer. The OMNIFIT frame has no equivalent, so an independent motion measure exists for the 27 SOSO recordings only. The layout is inferred from the frame, not documented. As a check, the vector magnitude should be nearly constant and equal to gravity, and in all 27 recordings it is. The median magnitude per recording ranges from 16,441 to 17,201 counts (cohort median 16,778), and the interquartile range within a recording is at most 399 counts. That corresponds to 1.00–1.05 g if the sensor is configured for a ± 2 g range at 16,384 counts per g. We have not confirmed that configuration, so accelerometer variability is used only as a relative, within-recording measure of movement.

The preprocessing sources also branch on the product, and the two paths are not equivalent. The branch is selected from a `bluetooth_type` field in each session's metadata, defaulting to the SOSO path when the field is absent. On the EEG side both paths apply the same Butterworth stages, z-score outlier removal and spline interpolation, but the OMNI path then divides each channel by its own standard deviation over the whole recording after subtracting that channel's mean, which the SOSO path does not do. On the PPG side the SOSO path filters and cleans the stored channel, while the OMNI path first subtracts a one-second (250-sample) centred moving average from the raw ADC values, then filters, cleans, interpolates and applies a final scale conversion.

The consequence of the channel-wise step is not a simple rescaling of the quantities we analyse. Band power is computed separately for each channel and the two are then averaged, so dividing each channel by its own standard deviation scales that channel's power by the inverse square of it: the average becomes $\frac{1}{2}(P_1/\sigma_1^2 + P_2/\sigma_2^2)$ rather than $\frac{1}{2}(P_1 + P_2)$. Whenever the two channels differ in standard deviation, their relative contribution to the averaged band power changes, and with it the contribution to any composite built on that average. We did not quantify the effect of this on the reported associations.

For the one session whose cleaned signals remain on local storage, the stored EEG is consistent with the channel-wise normalization implemented in the OMNI pathway—its metadata carries `bluetooth_type` OMNI, and its two cleaned channels have near-zero means and unit standard deviations to floating-point precision over its 1,136,331 samples—but that consistency does not establish the complete execution history, including which code version and PPG preprocessing steps were used. We cannot confirm the execution history for all 48 recordings. The stored metadata and cleaned files record no branch name or code version, the analysis batch reads already-preprocessed files so its logs say nothing about the branch, and we did not re-run preprocessing or retrieve the original execution logs for

this revision. The product assignment in our records is a device grouping and is not a substitute for that provenance. We therefore do not claim that the two products received identical preprocessing, and we do not claim to have audited which path ran for each session.

Assignment of participants to products was not randomized; it followed which units were free. Both were present on both recording days (3 SOSO / 2 OMNIFIT on 25 November, 24 / 19 on 27 November), so product is crossed with recording day rather than confounded by it, but it remains confounded with the participant.

S1.2 Electrical specification and its consequences

The device records mono-polar, with reference and ground carried on a clip attached to the right earlobe; the PPG sensor is built into that same ear clip and operates in transmission mode, so EEG and PPG are co-sampled at 250 Hz from a single unit. The frontal electrodes are dry metal contacts requiring no conductive gel or skin preparation; the manufacturer does not publish the contact alloy. Dry contacts carry a higher and less stable electrode–skin impedance than wet Ag/AgCl laboratory electrodes, which is one reason we treat these recordings as a physiological reference of limited precision.

The published specification is an EEG passband of 3–41 Hz at -3 dB, an input range of ± 590 µV at a resolution of 0.036 µV, internal noise of at most 0.8 µV$_{\mathrm{rms}}$, a pulse-rate measurement range of 30–200 beats min^{-1}, and an inter-beat-interval precision of 4 ms. Two consequences bear on the analyses. First, the hardware passband is narrower than the 3–50 Hz software filter applied in preprocessing, so the upper end of the analysed gamma band (40 Hz) lies close to the published upper cutoff—below it, but near it—while the software filter passes frequencies the instrument has already attenuated. Second, the 4 ms interval quantization is a floor on the precision of the RMSSD and MeanNN indices.

These are the manufacturer's current published figures. Other peer-reviewed work describing the same model reports a 3–43 Hz band, a ± 393 µV input range, input noise below 0.6 µV$_{\mathrm{rms}}$ and 15-bit resolution. We do not know which hardware revision each set of figures describes. Under either figure the analysed gamma edge at 40 Hz is below the stated corner rather than beyond it, and because we did not measure the frequency response of the units used here we cannot quantify the attenuation inside the analysed band from these specifications alone. What we do is treat gamma as the band most exposed to that uncertainty and use it in no primary analysis, rather than assert that it is the least accurate of the four.

S1.3 Product comparison

No statistically significant between-product differences were detected in the coupling measures (Table S1). One derived quantity does differ, in the 0.3–1.2 Hz re-derivation of Section 3.5: the median inter-beat interval is 840 ms on the SOSO units and 805 ms on the OMNIFIT units (Mann–Whitney $p = 0.009$). We do not attribute that to the instrument—product is confounded with participant, a 35 ms difference in median interval is well within the between-person range, and 21 against 27 unrandomized assignments cannot separate the explanations. In the wider-band re-derivation, the medians were 742 and 747 ms, and no statistically significant between-product difference was detected ($p = 0.92$). These exploratory comparisons do not establish whether the narrow-band difference reflects processing, participant characteristics, or device-related factors. It is recorded because it is one of two comparisons in which a between-product difference reached nominal significance; the other is the PPG pass fraction below. The interval-reliability problem of Section 3.5 is common to both: in the same narrow-band re-derivation, RMSSD falls in the physiologically expected range for 10 of 27 SOSO and 5 of 21 OMNIFIT participants, a difference that was not statistically significant (Fisher's exact $p = 0.37$).

Signal quality also differs by product. The median per-subject pass fraction was 99.2% on the SOSO units and 97.3% on the OMNIFIT units (Mann–Whitney $p = 0.013$), while median band agreement was 0.952 and 0.959 ($p = 0.98$). Flat windows were rare on both apart from the one SOSO recording whose channel never left the end of its range: 0.0% of the other SOSO windows and 0.1% of OMNIFIT windows. The OMNIFIT recordings have no accelerometer, so the movement association of Section 3.5 rests on the SOSO units alone. The difference in pass fraction is subject to the same caveat as every comparison here,

since product is confounded with participant.

We analysed the cohort jointly, while acknowledging that these comparisons do not establish equivalence between products and that product assignment was confounded with participant. A study designed to compare them would randomize the assignment, which this one did not.

Table S1: Comparison of the two headband products. Group means are Fisher-z averages of per-subject correlations; counts are subjects reaching nominal $p < 0.05$ under the circular-shift test. No statistically significant between-product differences were detected in the coupling measures; the two comparisons reaching nominal significance are the narrow-band re-derivation of the inter-beat intervals and the PPG pass fraction.

**Measure**	**SOSO** ($n = 27$)	**OMNIFIT** ($n = 21$)	**Test**
Within-subject arousal prediction, mean r	$+0.077$	$+0.078$	Welch $t = 0.01$, $p = 0.99$
subjects significant	7/27	6/21	Fisher $p = 1.00$
Alpha coupling under full control, mean r	$+0.026$	$+0.014$	Welch $t = 0.27$, $p = 0.79$
subjects significant	6/27	5/21	Fisher $p = 1.00$
Recording length, median windows	901	886	Mann–Whitney $p = 0.47$
Median inter-beat interval†	840 ms	805 ms	Mann–Whitney $p = 0.009$
same, wider band	742 ms	747 ms	Mann–Whitney $p = 0.92$
RMSSD in the 20–100 ms range†	10/27	5/21	Fisher $p = 0.37$
PPG pass fraction, median‡	99.2%	97.3%	Mann–Whitney $p = 0.013$
Band agreement, median‡	0.952	0.959	Mann–Whitney $p = 0.98$

†From the narrow-band (0.3–1.2 Hz) re-derivation of Section 3.5, not the stored pipeline values. ‡Median over subjects of the per-subject value from the quality screening of Section S1.5.

S1.4 Sampling rate, timing and alignment

Because the transport is a Bluetooth packet stream rather than a hardware-clocked acquisition, the realized rate carries packet-level jitter: the median is 249 samples s^{-1} with an interquartile spread of a few samples per second.

Windowing and feature extraction operate on sample index at the nominal 250 Hz, converting between time and index with the fixed constant rather than with the recorded timestamps. Over a 77-minute session a 1 samples s^{-1} rate error accumulates to about 18 s of index drift, so window boundaries in the biosignal stream and in the video stream are not guaranteed to correspond to the same instant to better than that. We did not quantify the realized error per session, and it is listed among the unresolved measurement limitations in Section 4.3 rather than presented as bounded. What is verified is the chronological separation of the training and test portions: the split verification described in Section S2.2 reads the window timestamps directly and does not depend on the index arithmetic.

One participant had been absent from the two re-processing passes because a verification step compared the re-extracted and archived series at zero time offset only, and that recording begins 450 s into the session; the participant is restored and all 48 are included throughout. This is separate from the participant excluded at the outset from the original 49 for having no video file, who remains excluded.

S1.5 PPG signal-quality screening

The screening of Section 2.2 was applied to the archived raw streams. They were decoded with the same parsers as the re-derivation of Section 3.5, not to the stored cleaned signals. In all 48 recordings the product identified from the frame format matches the device recorded for the participant. Windows are 30 s long and start every 5 s from sample 0, which is the grid of the stored features. In all 48 recordings the raw stream has the same length as the stored stream, so every scored window corresponds to a stored window by start time (43,923 windows).

Each recording was filtered as a whole before windowing, in two bands: 0.5–8 Hz for quality and 0.3–1.2 Hz for the analysis band. Both use the cascaded fifth-order zero-phase construction of Section 2.2. Because the filters are zero-phase and applied to the whole recording, a large artifact just outside a window

can extend into it; such windows are scored as they are, since the stored features were computed the same way. Per window:

- *Beats* were detected on the 0.5–8 Hz signal with the Elgendi detector in NeuroKit2.
- *Template correlation*: the mean, over the samples between the first and last beat, of the correlation of each pulse with the average pulse of that window. The template is built within the window rather than over the recording, so slow changes in pulse shape across a session of 46–110 minutes are not counted as artifact.
- *Feasibility*: the three rules of Orphanidou et al., applied to the beats of the whole 30 s window. Those rules were proposed for 10 s segments. Over 30 s the ratio rule is stricter, because a single missed or ectopic beat anywhere in the window fails it.
- *Flat fraction*: the share of raw samples lying in runs of at least 25 identical consecutive values (0.1 s).
- *Descriptors* (not used in the pass rule): the median of 3-s skewness (2-s overlap); the fraction of samples whose inter-beat interval is confirmed by a second detector (NeuroKit2 `ho2025`, a method proposed for the ECG); and the ratio of raw-signal power in 1–2.25 Hz to that in 0–8 Hz. The last is low whenever slow baseline drift dominates the raw stream, so it measures drift as well as artifact and is reported only as a descriptor.
- *Band agreement*: beats were re-detected with NeuroKit2 `ppg_process` on the 0.3–1.2 Hz signal and matched one-to-one to the 0.5–8 Hz beats within ± 150 ms. The result is reported as F1, $2m/(n_1 + n_2)$, where m is the number of matches and n_1, n_2 are the two beat counts. The product-dependent preprocessing, including the OMNI path's one-second moving-average detrend, is not re-executed, so the analysis-band beats here are a re-derivation and not the stored beats.
- *Movement* (SOSO only): the standard deviation of accelerometer magnitude within the window.

A window passes when template correlation is at least 0.86, all three feasibility rules hold, and the flat fraction is below 5%. Software: NeuroKit2 0.2.13, SciPy 1.17.1. The scoring script is `ppg_sqi.py`, the sensitivity analysis `ppg_sqi_sensitivity.py` and the random-mask control `ppg_sqi_random_mask.py`, all available with the other analysis scripts.

Two properties of the screen bound what a pass means. Template matching measures how alike the pulses in a window are, not whether they are pulses: artifact that is itself periodic at a plausible heart rate produces similar "pulses" and passes. On the recording whose channel never left the upper end of its range, template correlation on the filtered noise was 0.94, and only the flat-signal criterion rejected it. And because no electrocardiogram was recorded, passing establishes the absence of detectable contamination, not the correctness of the intervals.

S2 Statistical Implementation and Robustness

S2.1 The circular-shift test

Let x and y be two window-level series of length n from one subject, each z-scored. The observed statistic is the Pearson correlation $\rho = \frac{1}{n}\sum_i x_i y_i$. The null distribution is the set of correlations obtained by rotating y against x,

$$\rho_s = \frac{1}{n}\sum_{i=1}^{n} x_i \, y_{((i+s-1) \bmod n)+1}, \qquad s \in \{12, 13, \ldots, n-12\}, \tag{S1}$$

which is a set of exactly $n - 23$ values. We enumerate it in full rather than sampling from it: the whole set is obtained in one operation as the circular cross-correlation of the two series,

```
cc = np.fft.irfft(np.fft.rfft(y) * np.conj(np.fft.rfft(x)), n=n) / n
null = cc[12 : n - 12 + 1]
```

so no random draw enters the test and the same input always yields the same p-value. The minimum shift of 12 windows is 60 s at the 5 s step, twice the 30 s window length, and excludes rotations that leave the two series nearly aligned. For the held-out portions of Section 3.4 the enumeration contains 87 to 239 shifts across subjects; for the full-session analyses of Section 3.2 it contains 523 to 1283.

The p-value is the Phipson–Smyth estimator,

$$p = \frac{1 + \#\{s : |\rho_s| \geq |\rho|\}}{1 + K}, \qquad K = n - 23, \tag{S2}$$

which adds one to both numerator and denominator so that no p-value is reported as zero. Its lower bound is $1/(1 + K)$. For the held-out series K runs from 87 to 239, so the bound runs from $1/88 = 0.011$ down to $1/240 = 0.0042$: the subject with the shortest held-out series cannot be assigned a p below 0.011 however extreme the observed correlation.

What the test does and does not assume. A circular rotation preserves the *circular* autocorrelation of y at every lag exactly. It preserves the ordinary autocorrelation only approximately, because the rotation joins the end of the series to its beginning and creates one discontinuity. The null is therefore approximate, and it is approximate in a way that is not conservative by construction: we do not know the direction of the error. A subject with 88 usable shifts also has a null distribution estimated from 88 values, which is a coarse reference. We report the counts as descriptive at a nominal level for these reasons, and not as a corrected group-level inference.

An alternative null. We also ran a Fourier phase-randomized surrogate null (5000 surrogates, seed 0), which retains the amplitude spectrum of one series and randomizes its phases. This preserves the periodic autocovariance and keeps n fixed; it does not remove the periodic-boundary assumption. It is a second approximate null rather than an independent check on correctness, and where the two disagree we report both counts. Across the analyses in this paper the surrogate null never returns a higher count than the circular one and returns up to three fewer.

A truncating variant, and why we do not use it. A third variant discards the wrapped tail instead of rotating it, correlating only the $n - s$ overlapping positions. This removes the wrap discontinuity, but at the cost of a series whose length depends on the shift. It returns far fewer significant subjects than either null above: 3 of 48 for arousal and 2 of 48 for valence, against 13 and 11 under the circular test. We report that difference rather than omit it, and we neither treat it as the better test nor read it as a validation of the circular one.

Our reason for not using it is that its replicates are not comparable with the observed statistic: the correlation at shift s is computed on $n - s$ points while the observed ρ uses all n, and the larger sampling variance of the short overlaps at large s can widen the reference distribution. We have not established what that does to the variant's false-positive rate. Under serial dependence and possible non-stationarity we cannot show that its rejection rate is at or below the nominal level, so we do not describe it as conservative by construction, and the low counts are not themselves evidence of conservatism. The circular and phase-randomized nulls hold n fixed, which removes the length mismatch, and both instead carry the periodic-boundary approximation. We have no null here whose calibration we have verified, which is why every per-subject count in this paper is reported as descriptive.

S2.2 Train/test separation and the boundary purge

Windows are 30 s long and advance in 5 s steps, so each window shares raw samples with the five windows on either side of it. A chronological 80/20 cut at window index c therefore leaves the last five training windows overlapping the test period in raw samples. Those five windows are discarded, which is the smallest purge that achieves separation: the training portion ends at index $c - 5$ and the first test window begins at index c.

The separation was verified on the window timestamps rather than on the index arithmetic. For each of the 48 subjects, the script reads the last training window's end time and the first test window's start

time and reports their difference. The gap is $+0.0$ s for all 48 subjects—exactly zero, which is what a 30 s window at a 5 s step gives when five windows are removed—and no subject shows a negative gap. A negative value would indicate residual overlap.

S2.3 Boundary-gap sensitivity

Zero-phase filtering and the within-subject z-scoring of the reference are computed over the whole recording, so a purge that removes raw-sample sharing does not remove every dependency that crosses the split. Table S2 widens the gap beyond the minimum, discarding further training windows so that the two portions are separated by up to 600 s.

Table S2: Within-subject arousal prediction as the gap between the training and test portions widens. "Extra windows" are training windows discarded beyond the five required for raw-sample separation. Group means are Fisher-z averages over 48 subjects; p is a one-sample t-test on the Fisher-z values; "significant" counts subjects at nominal $p < 0.05$ under the circular-shift test.

**Extra windows**	**Gap (s)**	**Group mean r**	$\boldsymbol{p}$	**Significant**
0 (primary)	0	$+0.078$	0.069	13/48
6	30	$+0.076$	0.082	12/48
12	60	$+0.081$	0.061	13/48
24	120	$+0.087$	0.049	11/48
60	300	$+0.073$	0.110	12/48
120	600	$+0.062$	0.168	9/48

The group mean does not fall monotonically as the gap widens, and the nominal p crosses 0.05 in both directions across the six conditions while the training set shrinks. We interpret this as instability of a small effect under a shrinking sample rather than as evidence that a residual dependency is or is not present: the conditions differ in training size as well as in gap, and the design does not separate the two. The primary row is the one reported in the main article, and it is the least favourable of the first four.

S2.4 The composite construction

The arousal composite is z-scored over the whole session, which means the target the model is evaluated against uses statistics computed partly from the test period. Re-normalizing the two components on training rows only defines a *different* target, not a corrected version of the same one, because $z(\beta)-z(\mathrm{RMSSD})$ under two different normalizations is not an affine transform of itself: the two components are rescaled by different factors.

We verified the distinction directly. Rescaling the constructed composite by training-window statistics changes the reported correlation by 2×10^{-15}, since Pearson r is invariant to affine transformation of either variable. Normalizing the components separately changes the correlation substantively, to $+0.130$ ($p = 0.010$, 14/48 significant, 12 positive). Table S3 reports both. Forty-five of 48 subjects receive the same significance verdict under the two constructions. We do not read the difference between the rows as the size of a leakage bias, because the rows do not estimate the same quantity.

Table S3: The within-subject analysis under two constructions of the reference composite ($N = 48$). The model, the feature set, the split and the test are identical; only the target differs.

Reference composite	Question	Group mean r	Sig.	Positive
Normalized over the session (as defined)	retrospective	$+0.078$ ($p = 0.069$)	13/48	11/48
Components normalized on training rows	forward	$+0.130$ ($p = 0.010$)	14/48	12/48

The two targets differ per subject, so the rows are not a corrected and uncorrected version of one quantity and the difference between them does not measure a bias. Forty-five of 48 subjects receive the same significance verdict under both. Neither row is a fully independent prospective validation: zero-phase filtering over the whole recording crosses the split in both (Section 2.6 of the main article).

S2.5 Non-overlapping windows

Restricting the analysis to every sixth window removes raw-sample sharing between analysed windows entirely, at the cost of five sixths of the rows. Down-sampling also changes what a rotation of a given number of rows means in time: after it the rows are 30 s apart, so the twelve-row minimum used in the primary analysis would rotate by 360 s rather than 60 s. The minimum is therefore recomputed from the row spacing, at two rows, so that the 60 s criterion is the same in both runs. Under full geometric control the alpha count is then 6 of 48, against 11 of 48 in the primary analysis.

At the 360 s minimum the count is 7 of 48, differing in one subject. We report the 60 s figure because it holds the test's criterion fixed while the row spacing changes, and not because of which count it produces; the comparison is a check on window overlap only, and it does not remove the autocorrelation of the underlying signals, which the circular-shift null is there to address.

S2.6 Sensitivity to the arousal composite

Table S4: Alternative arousal ground-truth definitions evaluated in the sensitivity analysis. All quantities are within-subject z-scored before correlation.

ID	Definition	Interpretation
A0	$z(\beta) - z(\mathrm{RMSSD})$	Primary central + autonomic
A1	$z(\beta)$	Cortical only
A2	$-z(\mathrm{RMSSD})$	Autonomic only
A3	$z(\mathrm{MeanNN})$	Cardiac interval (inverse of rate)
A4	$z\big(\frac{\beta}{\alpha+\theta}\big)$	Vigilance ratio
A5	$-z\big(\frac{\theta}{\alpha}\big)$	Task engagement (inverted)
A6	$z\big(\frac{\beta+\gamma}{\alpha+\theta}\big)$	Broadband cortical shift

Table S5: Pupil (ID_mean) $\leftrightarrow$ alternative arousal ground-truth definitions ($N = 48$). Sig$^+$/Sig$^-$: number of subjects with within-subject $p < 0.05$ and positive/negative r.

GT	Definition	Mean r	95% CI	p	Mean $\lvert r\rvert$	Sig$^+$/Sig$^-$
A0	$z(\beta) - z(\mathrm{RMSSD})$ (primary)	+0.033	$[-0.014, +0.081]$	0.178	0.131	19/13
A1	$z(\beta)$	+0.019	$[-0.043, +0.079]$	0.556	0.158	17/12
A2	$-z(\mathrm{RMSSD})$	+0.030	$[-0.015, +0.074]$	0.198	0.120	20/9
A3	$z(\mathrm{MeanNN})$	−0.069	$[-0.134, -0.003]$	**0.046**	0.189	13/25
A4	$z\big(\beta/(\alpha+\theta)\big)$	−0.137	$[-0.203, -0.069]$	**< 0.001**	0.219	9/32
A5	$-z\big(\theta/\alpha\big)$	+0.061	$[+0.010, +0.111]$	**0.023**	0.147	25/12
A6	$z\big((\beta+\gamma)/(\alpha+\theta)\big)$	−0.147	$[-0.214, -0.079]$	**< 0.001**	0.222	9/30

The group-level ocular–composite coupling changes sign with the definition of the composite: the vigilance ratio (A4), the broadband cortical shift (A6) and the cardiac-interval index (A3) give negative group means, while the inverted theta–alpha ratio (A5) gives a positive one. Mean $|r|$ lies between 0.12 and 0.22 throughout. A group coefficient computed against "arousal" therefore cannot be reported without naming the composite.

Two cautions about reading this table. First, the raw correlations between the ocular measure and individual EEG bands are unchanged under every definition, because no composite enters their computation; that is an arithmetic identity, not evidence that those correlations are robust. Second, the seven definitions are not independent—A0, A1 and A2 share components, and A4 and A6 share a denominator—so the table shows how the sign depends on the formulation, not seven separate tests.

S2.7 The PPG-free ablation

Table S6: Section 3.4 of the main article repeated against an arousal reference containing no PPG ($N = 48$; exact circular-shift test over the enumerated shift set). Only the reference and the feature set change; the model, the 80/20 temporal split and the test are those used above.

Reference	Features	Group mean r	Sig. subjects	Positive
A0 $\frac{1}{2}[z(\beta) - z(\text{RMSSD})]$	all 13	$+0.078$	13/48	11/48
A1 $z(\beta)$, no PPG	all 13	$+0.248$	16/48	15/48
A1 $z(\beta)$, no PPG	no head pose (10)	$+0.223$	14/48	14/48
A1 $z(\beta)$, no PPG	ocular only (4)	$+0.174$	14/48	13/48

Repeating the within-subject analysis against a reference containing no PPG—$z(\beta)$ alone—and again with the head-pose features removed and with the ocular features alone, keeping the model, split and test unchanged, gives higher counts and group means than the primary composite (16, 14 and 14 of 48 significant; 15, 14 and 13 positive; group means $+0.248$, $+0.223$ and $+0.174$). The primary result therefore does not rest on the RMSSD term, on the facial features, or on the head-pose predictors. It does not follow that what remains is free of movement or of viewing geometry: the four ocular features are in raw image coordinates, and removing head-pose *predictors* does not remove the geometric variation inside `ID_mean` itself. We keep the pre-defined composite as the primary reference and report these as sensitivity checks, not as a better result to substitute for it.

S2.8 Sensitivity to PPG signal quality

Table S7: PPG signal quality and arousal prediction per subject, ordered by the fraction of windows passing screening; subjects are not identified. Agreement is the median per-window F1 between beats found in the analysis band and in the 0.5–8 Hz band. Retained is the fraction of windows kept when screening also requires agreement ≥ 0.95. r is the held-out correlation of Section 3.4 on all windows, on screened windows, and on screened windows whose bands agree; * circular-shift $p < 0.05$; — subject excluded from that condition (fewer than 60 training or 40 test windows).

	Product	Windows	Pass	Agreement	Retained	r all	r screened	r agree
1	SOSO	901	0.0%	0.44	0%	−0.10	—	—
2	SOSO	1,310	0.0%	0.14	0%	−0.07	—	—
3	OMNI	621	33.7%	0.89	11%	−0.26	−0.40	—
4	OMNI	1,292	43.1%	0.94	33%	+0.30*	−0.09	—
5	OMNI	835	83.1%	0.88	13%	−0.22	−0.21	—
6	SOSO	808	83.3%	1.00	80%	+0.03	+0.43	+0.34
7	OMNI	1,192	83.8%	0.82	5%	−0.33	−0.28	—
8	OMNI	1,019	90.7%	0.93	31%	+0.32	+0.34	—
9	OMNI	888	91.2%	1.00	84%	−0.01	+0.26	+0.25
10	OMNI	801	91.4%	0.95	42%	+0.20	+0.11	+0.38*
11	OMNI	888	92.3%	0.80	8%	+0.11	−0.40	—
12	OMNI	1,133	94.4%	0.96	52%	+0.40*	+0.39*	−0.17
13	OMNI	1,172	95.0%	0.99	75%	+0.03	+0.16	+0.15
14	SOSO	903	95.9%	0.79	0%	−0.27	−0.25	—
15	SOSO	927	96.0%	1.00	94%	−0.02	+0.13	+0.14
16	SOSO	707	97.3%	0.76	0%	−0.02	+0.13	—
17	OMNI	1,012	97.3%	0.94	37%	+0.17	+0.01	−0.23
18	OMNI	709	97.5%	1.00	84%	+0.67*	+0.55*	+0.33
19	SOSO	1,162	97.8%	0.98	78%	+0.67*	+0.63*	+0.57*
20	SOSO	1,071	97.9%	0.81	5%	−0.06	−0.16	—
21	SOSO	1,176	97.9%	0.93	33%	+0.23	+0.21	−0.08
22	OMNI	889	98.0%	0.93	41%	+0.07	+0.03	−0.11
23	OMNI	809	98.1%	0.95	50%	−0.14	−0.17	—
24	OMNI	1,060	98.4%	0.99	82%	+0.23*	+0.24	+0.21
25	SOSO	873	98.5%	0.88	7%	+0.31*	+0.26*	—
26	SOSO	1,153	98.5%	0.88	20%	−0.02	−0.12	—
27	OMNI	904	98.7%	0.97	67%	−0.39*	−0.42*	−0.49
28	SOSO	779	98.7%	1.00	97%	+0.28*	+0.25	+0.28
29	OMNI	1,191	98.9%	0.99	75%	−0.11	−0.03	−0.14
30	SOSO	1,170	99.1%	0.92	26%	+0.27*	+0.28*	+0.19
31	OMNI	661	99.1%	1.00	95%	+0.29*	+0.32*	+0.12
32	SOSO	918	99.2%	0.98	84%	−0.31	−0.31	−0.23
33	SOSO	940	99.5%	0.95	45%	−0.29	−0.25	+0.18
34	SOSO	954	99.5%	1.00	93%	−0.28	−0.22	−0.26
35	SOSO	963	99.6%	0.94	37%	+0.13	+0.14	−0.03
36	OMNI	573	99.8%	0.99	80%	−0.02	−0.02	−0.08
37	SOSO	973	99.9%	0.99	93%	+0.13	+0.13	+0.02
38	OMNI	549	100.0%	0.98	82%	−0.11	−0.11	−0.01
39	OMNI	551	100.0%	0.97	64%	+0.29	+0.29	−0.25
40	SOSO	653	100.0%	0.94	38%	+0.32	+0.32	−0.26
41	SOSO	668	100.0%	0.97	78%	+0.05	+0.05	+0.03
42	SOSO	687	100.0%	1.00	100%	−0.53*	−0.53*	−0.53*
43	SOSO	813	100.0%	1.00	96%	+0.39	+0.39	+0.30
44	SOSO	827	100.0%	0.99	90%	+0.20	+0.20	+0.25
45	SOSO	836	100.0%	0.95	55%	−0.00	−0.00	+0.33
46	SOSO	837	100.0%	1.00	100%	+0.33*	+0.33*	+0.33*
47	SOSO	869	100.0%	0.95	54%	+0.48*	+0.48*	+0.56*
48	SOSO	1,296	100.0%	1.00	99%	+0.05	+0.05	+0.08

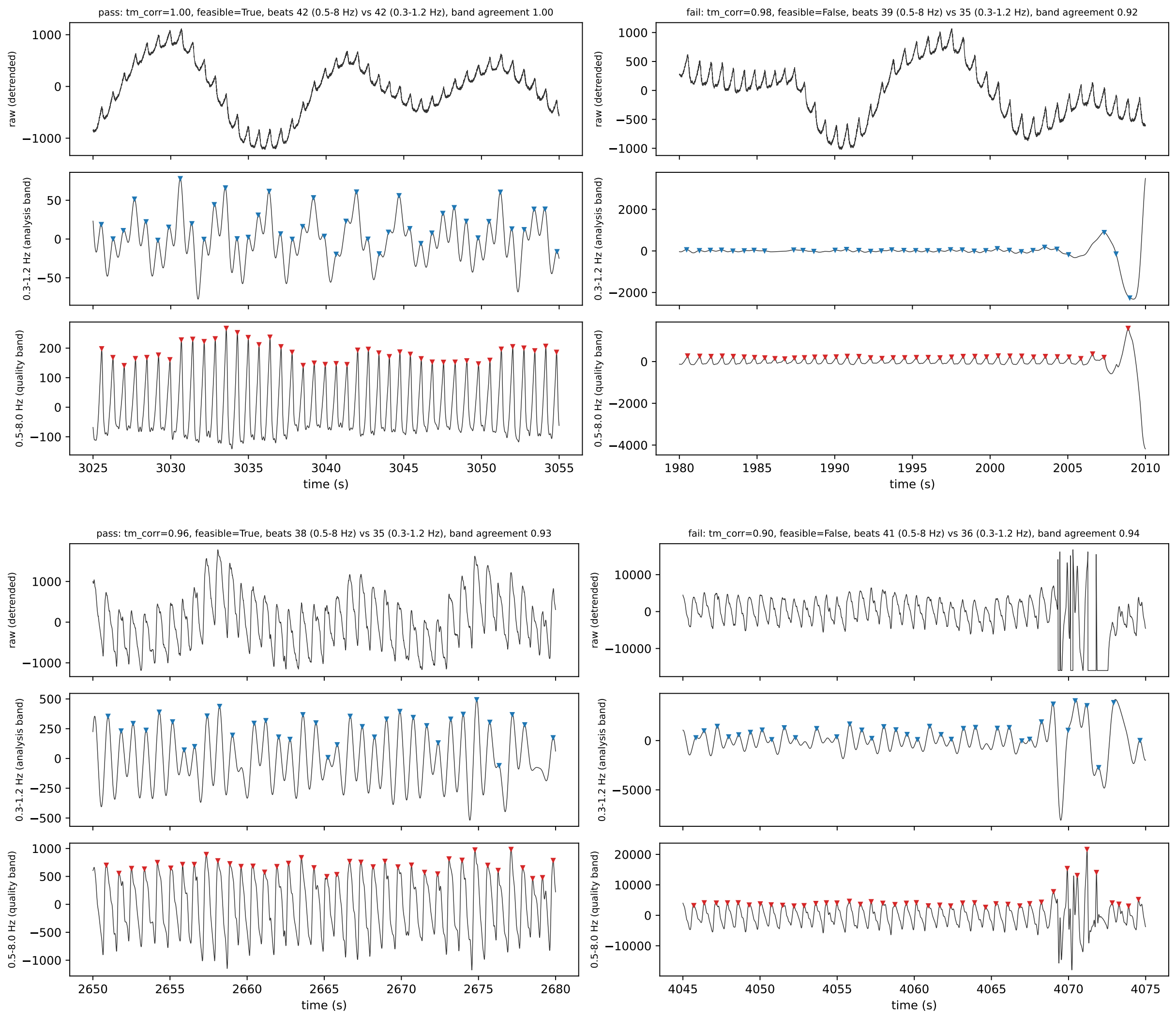

Figure S1: Raw (detrended), analysis-band (0.3–1.2 Hz) and 0.5–8 Hz PPG for one passing (left) and one failing (right) 30 s window from each product: a SOSO participant (top) and an OMNIFIT participant (bottom). Each participant is the one whose pass fraction is closest to that product's median (99.2% and 97.3%). Within each participant, each window is the one at the median template correlation of its group, not the clearest example. Triangles mark the beats found by the detector in each band. In the top-left window the analysis-band trace has lost the pulse shape that the 0.5–8 Hz trace keeps, although here the beats found in the two bands still coincide.

S3 The Intensity-Ratio Measure

The preceding analysis shows that the inter-landmark distance is dominated by camera geometry. That conclusion concerns one particular measurand, not the recordings themselves, and it invites a separate question: does the video carry pupil-related information that a measure not built on landmark distance could recover? We therefore re-processed the source recordings a second time to compute, for each frame and each eye, the ratio of mean grey level over a central disc (within 35% of the iris radius) to that over a surrounding annulus (55–90%),

$$C(t) \;=\; \frac{\overline{I}_{\mathrm{disc}}(t)}{\overline{I}_{\mathrm{annulus}}(t)}, \tag{S3}$$

with specular highlights above the 95th percentile excluded. Both regions are sampled from the same eye in the same frame, so a ratio of their means is unchanged by any factor that scales both equally—a uniform multiplicative change in illumination, or a change in apparent size that rescales the two regions

together. That is the sense, and the only sense, in which C is insensitive to distance and lighting. It is not invariant to them in general: shadowing and oblique illumination change the two regions unequally, specular reflection is removed only by the percentile cut, and at 21 pixels across the iris the disc and annulus both contain mixed pixels whose composition shifts with apparent size. C also shares the source video with the landmark measure, so it is a second index computed from the same recordings rather than an independent measurement. What it does avoid is the pupil margin, so it is not bounded by the edge-localization limit of Section 2.4 in the same way. We treat it as an exploratory image-derived index rather than a calibrated measure. A dilating pupil darkens the disc relative to the annulus, so C is expected to vary *inversely* with pupil size. Across the cohort the median C was 0.896.

Two properties were checked before relating C to EEG. First, C is essentially independent of head movement: the mean within-subject correlation with the frame-to-frame coefficient of variation of face width was -0.086, and the magnitude-squared coherence between C and the head-pose signals in the pupillary-unrest band (0.05–0.5 Hz) did not exceed 0.17 in any subject, against a 0.3 criterion adopted for this check. Second, and less favourably, C is strongly related to eyelid aperture: the mean within-subject correlation with the eye-aspect ratio was $+0.489$, exceeding 0.3 in magnitude in 39 of 48 subjects. A descending lid casts shadow into the central disc and lowers C without any change in pupil size, so this term must be removed before C can be interpreted.

Table S8 reports the group-level correlation between C and EEG band power under the same cumulative controls used above, with eyelid aperture added. Three features of the result bear on the question posed at the start of this section. The alpha and theta associations survive the full control set, at -0.052 and -0.046 respectively. Individually, 13 of 48 subjects show alpha coupling that survives both the full control set and the circular-shift test used in Section 3.2 (an uncorrected Pearson test gives 30/48; the overlap inflates the count of significant subjects here as it does there, and leaves the correlations themselves unchanged). The sign is negative, which is the direction expected if C tracks pupil size inversely, and therefore agrees with the positive—if weak—association found for the landmark measure. The band profile is also informative: in frequency order the coupling magnitude is 0.046 (theta), 0.052 (alpha), 0.043 (beta) and 0.019 (gamma), so it peaks in the alpha band and falls away into beta and gamma rather than rising with frequency. Movement and myogenic contamination produce the opposite profile, growing into the higher bands, which is the pattern that led us to reject an earlier within-window variability measure. We note that the profile is not monotonic and we do not treat it as a formal test; it is a qualitative check that the coupling does not carry the artifact signature. The uncontrolled rows illustrate why the eyelid term matters: before it is removed the gamma band carries a positive association—significant once movement is controlled—which disappears entirely once lid aperture is taken out.

Table S8: Group-level correlation between the intensity ratio C and EEG band power under cumulative control ($n = 48$; Fisher-z one-sample test). Negative values are expected, since C varies inversely with pupil size. Each level uses the complete cases for exactly the covariates it names, and the group correlation and the per-subject test at a level are computed from the same residuals; one subject has three windows without a luminance sample, which is why the two lower rows use 744 of that subject's 747 windows. The p-values are nominal and uncorrected: four bands were examined at five levels of control, and no cell would survive a Bonferroni correction over the four bands at the full-control level.

**Control**	**Theta 4–8 Hz**	**Alpha 8–13 Hz**	**Beta 13–30 Hz**	**Gamma 30–40 Hz**
None	-0.061*	-0.073*	-0.015	$+0.060$
+ movement	-0.039	-0.054	$+0.007$	$+0.084$*
+ movement, eyelid	-0.079*	-0.094*	-0.052*	$+0.003$
+ movement, eyelid, luminance	-0.073*	-0.087*	-0.057*	-0.006
+ movement, eyelid, luminance, head pose	-0.046*	-0.052*	-0.043*	-0.019
Subjects individually significant under full control				
Pearson $p < 0.05$	31/48	30/48	30/48	29/48
Circular-shift $p < 0.05$	8/48	**13/48**	10/48	8/48

* $p < 0.05$.

We are deliberate about what this does and does not establish. It is not a validation of pupillometry: no reference pupil diameter was available in this dataset, so C has not been compared against a ground-truth measurement, and we make no claim about its accuracy in millimetres. What the analysis supports is narrower—that these recordings contain ocular intensity variation whose association with EEG survives removal of eyelid aperture, luminance and head pose, is in the direction predicted for pupil size, and does not show the frequency profile that movement and myogenic contamination would produce. None of that rules out a movement path: the coherence and correlation checks above bound a linear relationship with the head-pose signals we recorded, and a nonlinear or unmeasured path remains possible. The band profile is a qualitative check and not a test that excludes electromyographic contamination. The effect is small—a typical within-subject association of about five hundredths—and we do not convert it into a proportion of variance, since a mean of per-subject correlations does not square into one. The per-subject results are the more informative ones. Establishing whether C recovers pupil diameter in any metric sense requires simultaneous infrared pupillometry, which we identify as the necessary next step (Section 4.3).

S4 Secondary and Exploratory Analyses

S4.1 Facial features at the group level

Table S9: Facial features extracted as action unit (AU) proxies.

**Feature**	**Landmarks**	**AU Proxy**
EAR (eye aspect ratio)	159/145, 386/374, 33/133, 362/263	Eye openness
Brow inner distance	107 $\leftrightarrow$ 336 / face_w	AU4 (brow furrow)
Brow raise (L/R)	(eye_top.y − brow_top.y) / face_h	AU1/2
Mouth corner height	(nose.y − corner.y) / face_h	AU12/15
Mouth openness	(top_lip.y − bottom_lip.y) / face_h	AU25/26
Head yaw	(nose.x − face_center.x) / face_w	Head turn
Head pitch	(nose.y − face_mid.y) / face_h	Head tilt
Head roll	$\arctan$(eye_line)	Head roll

Three of the nine non-ocular features reached group-level significance against the biosignal-derived arousal index at a nominal level (Table S10); for valence none of the nine did. The associations are small, and two of the three are head-pose angles rather than expressive features, which is consistent with movement contributing to them. The proper test of whether facial dynamics carry arousal information within individuals is the per-subject model of Section 3.4, where these features enter as predictors; the ablation in Section S2.7 shows that removing them does not remove the within-subject result.

Table S10: Facial features ↔ EEG/PPG-derived arousal and valence (group level, Fisher z-test).

**Feature**	r **(Arousal)**	p	r **(Valence)**	p	**Interpretation**
`face_ear_mean`	$+0.070$	**0.018**	-0.038	0.156	Eye openness
`face_head_pitch`	$+0.065$	**0.027**	-0.007	0.814	Head orientation
`face_head_roll`	-0.044	**0.040**	$+0.019$	0.385	Head tilt

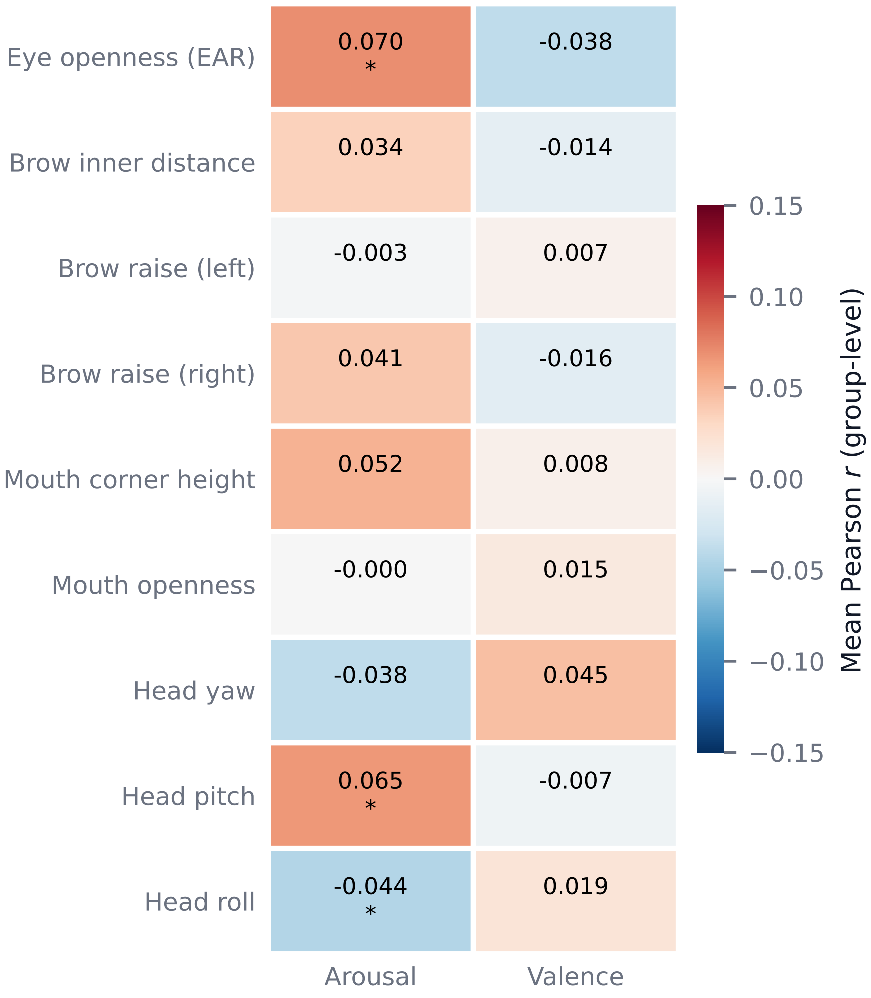

Figure S2: Group-level correlations between the nine non-ocular features and the biosignal-derived arousal and valence indices. Asterisks denote nominal significance: * $p < 0.05$, ** $p < 0.01$, *** $p < 0.001$, uncorrected across the 18 cells shown.

S4.2 A single-subject illustration

Figure S3 shows one subject's held-out segment: the biosignal-derived reference and the camera-derived prediction, for the subject the earlier draft of this work used as an illustration. It is drawn from the reported

condition—the same model, the same chronological 80/20 split and the same five-window purge—so the traces and the statistics below them are the ones the cohort analysis uses. The subject has 652 windows, of which 516 are used for training after the purge and 131 form the held-out segment.

Neither panel is individually significant under the circular-shift test ($r = +0.323$, $p = 0.119$ for arousal; $r = -0.294$, $p = 0.073$ for valence, over 108 admissible shifts). We show it as an illustration of what the two traces look like at this scale and not as a positive result; the subject was chosen for the earlier draft, and choosing a different one would give a different picture. The cohort distribution in Figure 3 of the main article is the summary that matters.

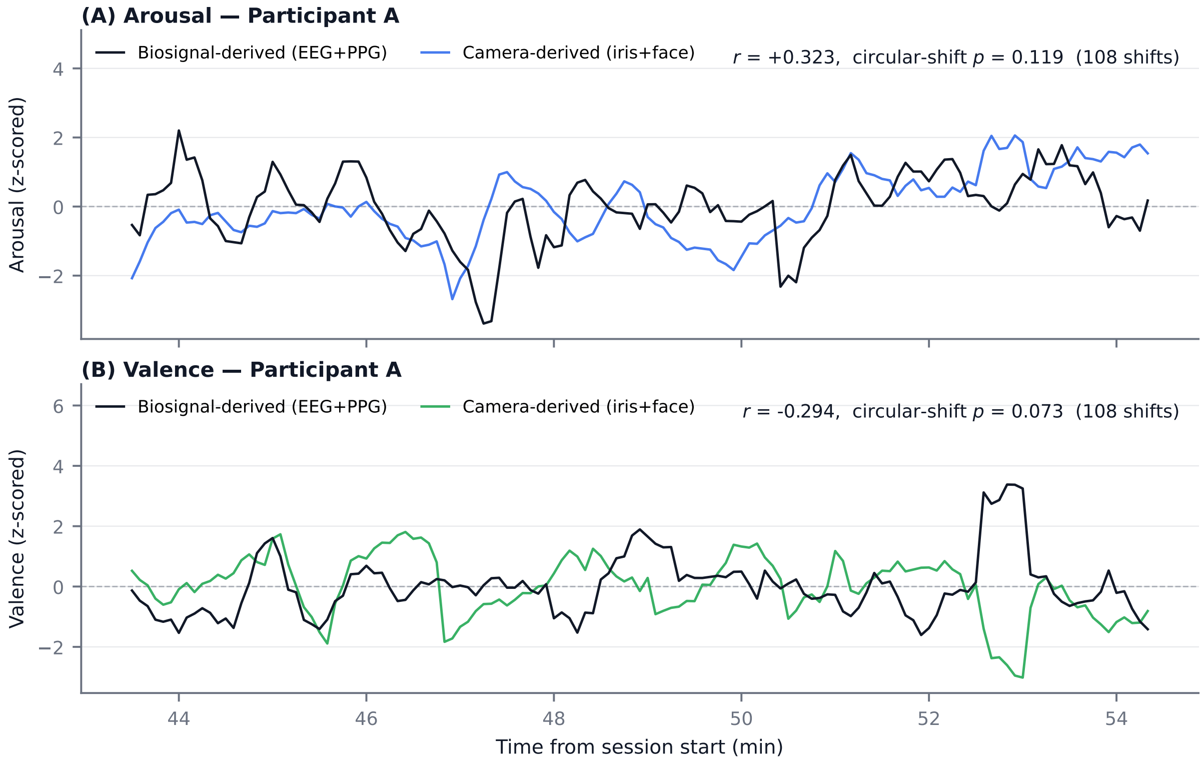

Figure S3: Biosignal-derived (black) and camera-predicted (coloured) (**A**) arousal and (**B**) valence for one subject, over the held-out final 20% of the session (131 of 652 windows). Both traces are z-scored. The model is the within-subject Ridge of Section 2.6, fitted on the first 516 windows after the five-window purge; the annotation gives the Pearson r and the circular-shift p-value over the segment shown.

S4.3 Cross-lagged and Granger analyses

As an auxiliary analysis, bivariate Granger causality (VAR($p = 3$) F-test) was computed between `ID_mean` and each biosignal target. The proportion of subjects with an F-test p below 0.05 was 21 of 48 for theta, 20 of 48 for alpha, 17 of 48 for beta, 14 of 48 for RMSSD, and 9 of 48 for HF (Figure S4).

We report these counts and nothing further from them. Three limitations apply. The p-values are computed across windows that overlap by 25 s; the autoregressive terms absorb serial dependence in the target but not the overlap between windows, so the nominal level is not the realized one and we do not compare the counts against a 5% expectation. We did not test the residuals for whiteness or the series for stationarity, which a Granger specification requires, so we do not present the counts as a valid test of precedence. And the analysis operates on 30 s windows advancing in 5 s steps: its temporal resolution is tens of seconds, so it cannot address the sub-second lead reported in the neurophysiological literature—which for spontaneous pupil events runs from cortex to pupil rather than the other way round—and we draw no connection between the two. The analysis is a descriptive temporal-pattern check, separate from the corrected evidence in the main article.

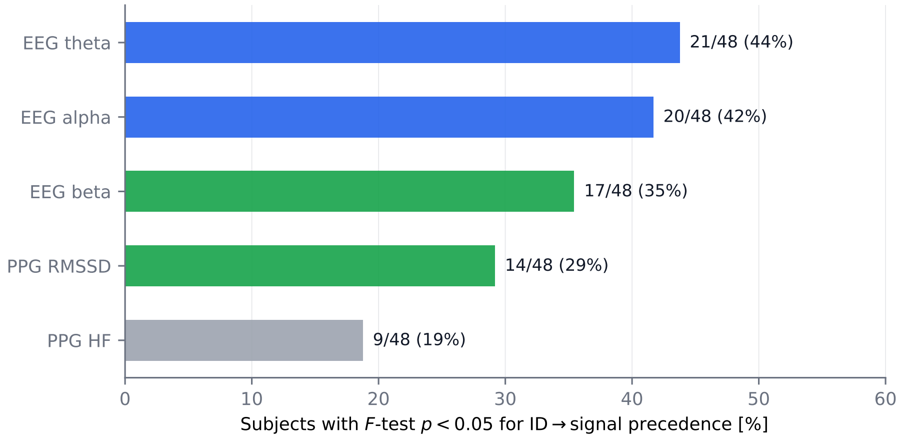

Figure S4: Proportion of subjects whose VAR(3) F-test for precedence of `ID_mean` over each biosignal reaches nominal $p < 0.05$. No chance-level reference is drawn: the windows overlap by 25 s, so 5% is not the expected proportion under the null. See the three limitations in Section S4.3.

S4.4 Group means and coupling magnitude before geometric normalization

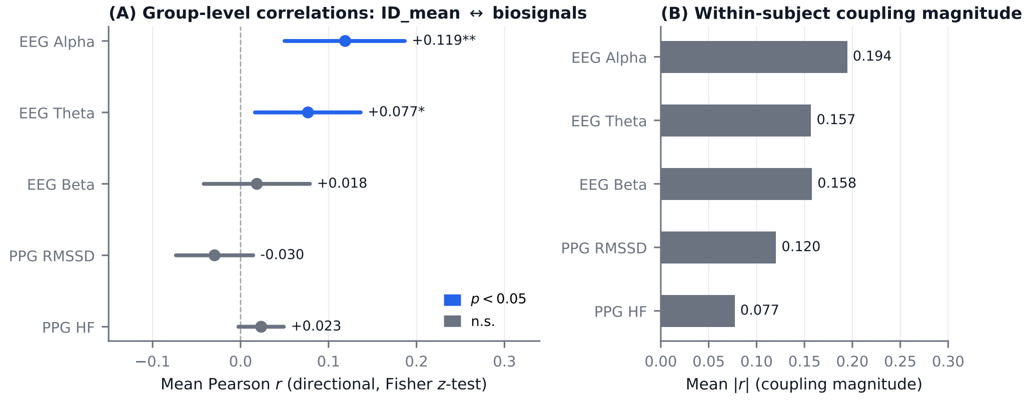

Figure S5: The raw measure, before geometric normalization. **(A)** Group-level correlation between `ID_mean` and each biosignal index: the point is the Fisher-z mean of the 48 per-subject correlations and the bar is its 95% confidence interval, computed as $\tanh(\bar{z} \pm 1.96\, s_z/\sqrt{48})$ over subjects. The interval is therefore an interval on the group mean across subjects, not a per-subject interval across windows, and window overlap does not enter it; blue marks $p < 0.05$ in the one-sample t-test on the Fisher-z values. These are the same five rows as Table 2 of the main article. **(B)** Mean of the *absolute* per-subject correlations for the same signals. The bars are one colour because a mean $|r|$ is a descriptive magnitude with no significance test attached; it exceeds the signed mean in every band because the per-subject correlations differ in sign.

Panel B is the quantity referred to in Section 4.1: for beta the signed mean is $+0.019$ while the mean $|r|$ is 0.158, because 28 subjects are positive and 20 negative. That gap is arithmetic, and it is not evidence of opposing physiological couplings. One caution about reading the magnitudes themselves: a mean of absolute correlations is positive even when every underlying association is zero, because $|r|$ of an estimate that scatters around zero is positive by construction, so the height of a bar in panel B is not a lower bound on any real coupling. This is a separate matter from the window overlap: overlap makes an ordinary p-value wrong, because successive windows are not independent draws, and that is what the test of Section 2.7 addresses. Neither overlap nor the circular-shift test changes the observed correlation itself; the correction is to the significance statement, not to the coefficient.

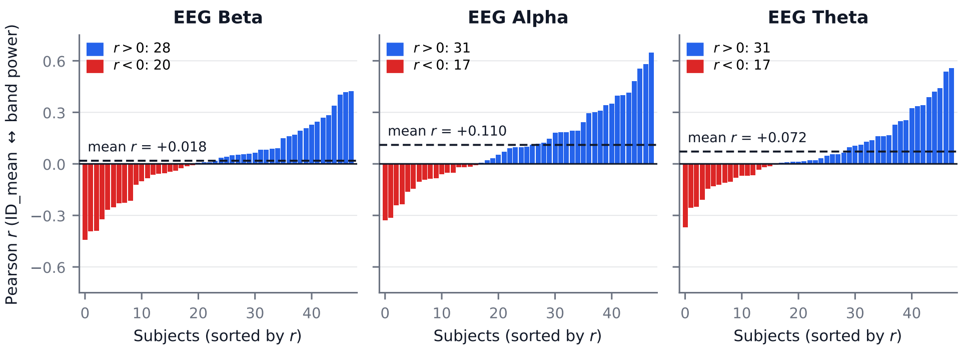

Figure S6: Distribution of per-subject correlations between `ID_mean` and each biosignal target, before geometric normalization. The spread in both directions, rather than the location of any distribution, is the feature of interest.

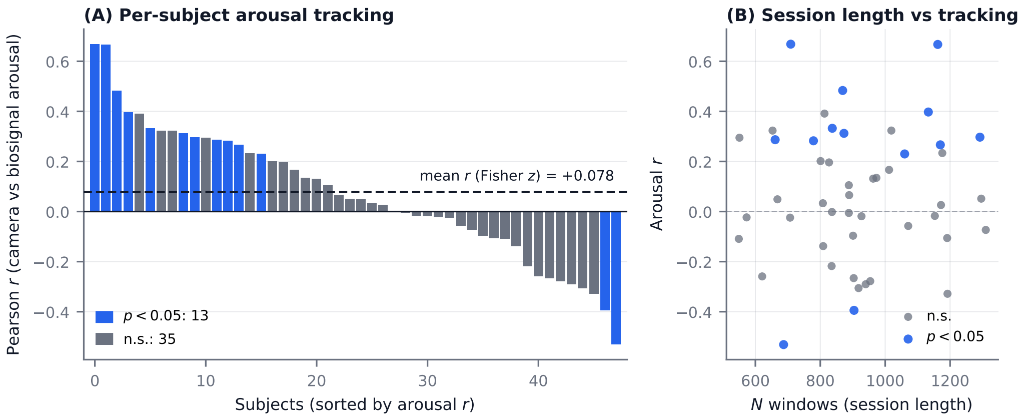

Figure S7: Per-subject held-out accuracy for the arousal index under the reported condition, ordered by value, with the subjects reaching nominal significance under the circular-shift test marked. Drawn from `results/ridge_permutation.csv`, the same file as Table 4 of the main article.

S4.5 Subjects with and without significant coupling

The two splits contain different subjects—11 of 48 retain alpha coupling under full geometric control, 13 of 48 show a significant within-subject arousal association—so their group comparisons are reported separately (Table S11). No comparison reaches significance. With 11 or 13 subjects on one side, that is a failure to detect a difference and not evidence that the groups are alike: the intervals these tests would support are wide, and we did not compute the difference each test could have detected.

Table S11: Subjects reaching nominal significance compared with those not reaching it, for each of the two splits. Session length is the mean number of analysed windows (Welch t-test); age is the group mean in years (Welch t-test); sex is the count of female subjects in the significant group against 32 of 48 in the cohort (Fisher's exact test); the last row correlates age with coupling strength, $|r|$ for the alpha split and the signed held-out r for the arousal split. None of the six comparisons is significant, and none is corrected for multiplicity.

Comparison	Alpha coupling (11 vs 37)	Arousal association (13 vs 35)
Session length (windows)	896 vs 915, $p = 0.80$	932 vs 903, $p = 0.67$
Age (years)	28.4 vs 31.3, $p = 0.10$	30.1 vs 30.8, $p = 0.72$
Sex (female in group)	10 of 11, $p = 0.07$	9 of 13, $p = 1.00$
Age vs coupling strength	$r = +0.034$, $p = 0.82$	$r = +0.062$, $p = 0.68$

S4.6 Cross-subject models

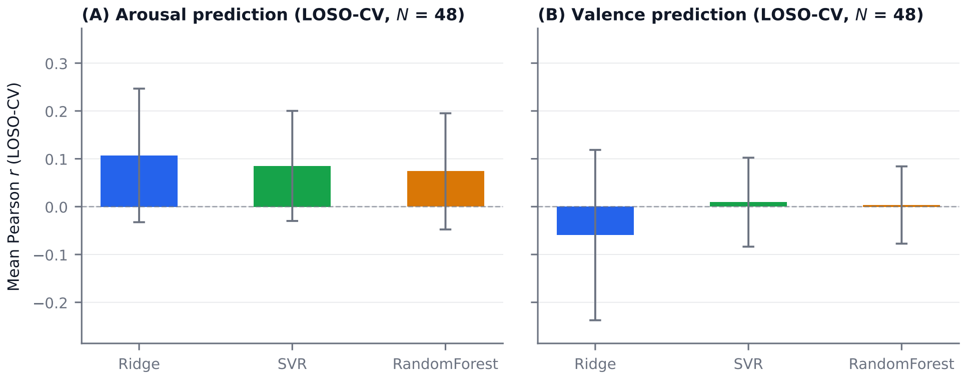

Figure S8: Leave-one-subject-out accuracy for three model families. The features are normalized within subject over the whole session before the fold is taken, so each held-out subject's own session statistics enter its features; the condition is disclosed in Section 3.4 and the values are not a prospective cross-subject accuracy.

Leave-one-subject-out cross-validation gave a group mean r of $+0.107 \pm 0.140$ for Ridge, $+0.085 \pm 0.115$ for a support-vector regressor and $+0.074 \pm 0.121$ for a random forest on the arousal index, and -0.059, $+0.009$ and $+0.003$ respectively on valence. Two conditions bear on how these are read. The within-subject feature normalization is computed over each subject's whole session including the held-out fold, so the numbers are not a prospective cross-subject accuracy. And the three model families were compared after the fact; we report all three rather than the best.

S5 An Untested Candidate Design for Per-User Calibration

This section describes a candidate approach in which monitoring is conditional on calibration performance, as discussed in the main article. No stage of the proposed procedure has been evaluated here, and this study does not establish whether participant-specific calibration improves performance over a population model on independent data.

The motivation is the heterogeneity in Section 3.4: the per-subject models fitted and evaluated within each session differ in accuracy in magnitude and in sign, and their group mean ($r = +0.078$, $p = 0.069$) was not statistically significant. That figure is the mean over *within-subject* models and is not a population model's accuracy; the cross-subject models are reported separately in Section 3.4, where leave-one-subject-out Ridge reaches $r = +0.107$ under the retrospective normalization condition stated there. We do not substitute one for the other, and this study ran no comparison that would establish which performs better on independent data. Prior work reports that clustering subjects and fitting cluster-specific models improves physiological emotion recognition over a single population model [1]; that is a comparison we did not run.

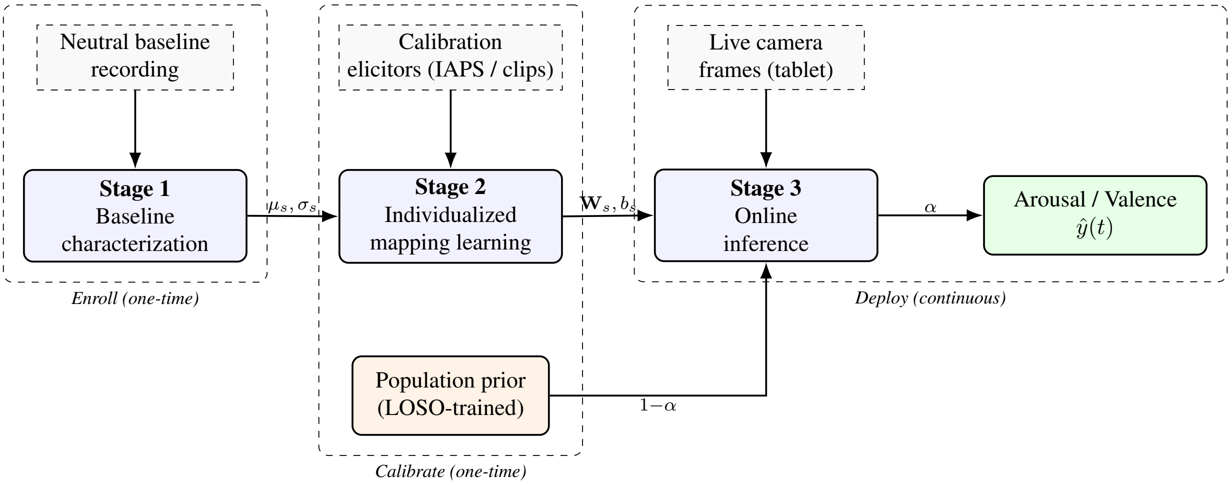

Figure S9: Proposed three-stage personalized calibration pipeline. Stage 1: per-subject normalization from a brief neutral baseline, where μ_s and σ_s are the mean and standard deviation of each camera feature for subject s, used to z-score incoming features. Stage 2: subject-specific linear mapping from camera features to valence–arousal targets, where $\mathbf{W}_s$ is the subject's feature weight vector and b_s the intercept (Equation (S4)), supplemented by a leave-one-subject-out (LOSO) population prior. Stage 3: convex combination of the personalized and population predictions, where $\alpha \in [0, 1]$ is the per-subject fusion weight ($\alpha = 1$ uses the personalized model only, $\alpha = 0$ the population prior only).

Stage 1—Baseline characterization. The user performs a short neutral rest recording (eyes open, passive viewing of a static neutral stimulus) while camera features are extracted. Per-subject means and standard deviations of the thirteen features are stored as that subject's normalization parameters $\{\mu_s, \sigma_s\}$ and used to z-score incoming features, converting raw MediaPipe outputs into subject-relative deviations. This removes a constant per-subject offset and scale. It does not address the problem identified in Section 3.2: face scale varies *within* a session with posture, and a single pair $\{\mu_s, \sigma_s\}$ estimated once at enrolment cannot remove a time-varying confound. Per-frame geometric normalization, as used in Section 3.2, would still be required and is a separate operation from this stage.

Stage 2—Individualized mapping. The user engages with a brief calibration battery of standardized affective elicitors spanning the valence–arousal plane, recorded alongside a short-term biosignal reference. A subject-specific linear mapping is fitted on the resulting paired sequences,

$$\hat{y}_s(t) = \mathbf{W}_s \cdot \mathbf{x}_s(t) + b_s, \tag{S4}$$

where $\mathbf{x}_s(t) \in \mathbb{R}^{13}$ is the normalized camera feature vector for subject s at time t, $\mathbf{W}_s \in \mathbb{R}^{1\times 13}$ the subject's weight vector, and $b_s \in \mathbb{R}$ an intercept absorbing a constant offset. Whether a per-subject fit generalizes to a later session is exactly what this study does not establish: the models reported in the main article were fitted and evaluated inside one session.

Stage 3—Online inference with a population fallback. This is a *second* candidate, and it is not the same design as the calibration gate described in Section 4.2 of the main article. The gate withholds monitoring from a user whose calibration accuracy falls below a threshold; the fallback below instead keeps producing an output for that user by leaning on a population model. They answer the same situation in incompatible ways—one declines to report, the other reports something else—and we do not specify how they would be combined, because neither has been evaluated. Both are listed so that a reader does not take either for a validated procedure. Under the fallback, incoming features are normalized with $\{\mu_s, \sigma_s\}$, mapped with $\{\mathbf{W}_s, b_s\}$, and combined with a population-level prediction,

$$\hat{y}(t) = \alpha\, \hat{y}_{\text{personal}}(t) + (1 - \alpha)\, \hat{y}_{\text{population}}(t), \tag{S5}$$

with $\alpha \in [0, 1]$ chosen per subject on held-out calibration data. The intent is graceful degradation for users whose calibration is uninformative rather than an accuracy claim.

What evaluating this would require. A prospective study with at least two sessions per subject, in which the per-user mapping and the acceptance threshold are fixed on the first session and the accuracy is measured on the second. Only such a design can produce a pass rate for a new user; the subgroup proportions in this paper were identified by inspecting the same held-out accuracies that summarise them and are not pass rates. The open parameters are the calibration duration, the minimal elicitor set, the fusion weight and the acceptance threshold; we have fixed none of them and report no value for any.

Two measurement improvements belong in such a study. A photometric sensor logging ambient illuminance at the eye would separate the pupillary light reflex from the residual more cleanly than the image-based grey level used here, which responds to exposure and gain as well as to scene illuminance. And a reference pupil measurement—infrared pupillometry recorded concurrently—is the only way to establish whether either the landmark measure or the intensity ratio of Section S3 recovers pupil diameter in any metric sense. A subjective measure recorded alongside would address the second link of the construct-validity chain that this study leaves untested.

Source files. Every number in this document is produced by the scripts named in the main article's data-availability statement. The circular-shift test is `src/analysis/circshift.py`; the within-subject analysis and its variants `src/analysis/ridge_permutation.py`; the split verification `src/analysis/verify_split.py`; the boundary sweep `src/analysis/boundary_sensitivity.py`; the composite check `src/analysis/verify_composite_leak.py`; and the single results file the manuscript is checked against `results/final_numbers.json`.

References

[1] Gutiérrez-Martín, L.; et al. Personalized Clustering for Emotion Recognition Improvement. *Sensors* **2024**.
