# Supplementary material for: Tablet-Camera Iris Landmarks and EEG/PPG-Derived Arousal Indices During Naturalistic Reading: Geometric Confounding and Exploratory Within-Subject Associations
Source: Sensors (Basel). 2026 Oct 14;26(20):6505. doi: 10.3390/s26206505 (PMC13709060; doi:10.3390/s26206505)
Supplement: Supplementary file 1 [file sensors-26-06505-s001.zip › Definitions/logo-updates-eps-converted-to.pdf]

Check for updates
