# Supplementary material for: Tablet-Camera Iris Landmarks and EEG/PPG-Derived Arousal Indices During Naturalistic Reading: Geometric Confounding and Exploratory Within-Subject Associations
Source: Sensors (Basel). 2026 Oct 14;26(20):6505. doi: 10.3390/s26206505 (PMC13709060; doi:10.3390/s26206505)
Supplement: Supplementary file 1 [file sensors-26-06505-s001.zip › figures/fig2_forest_plot.pdf]

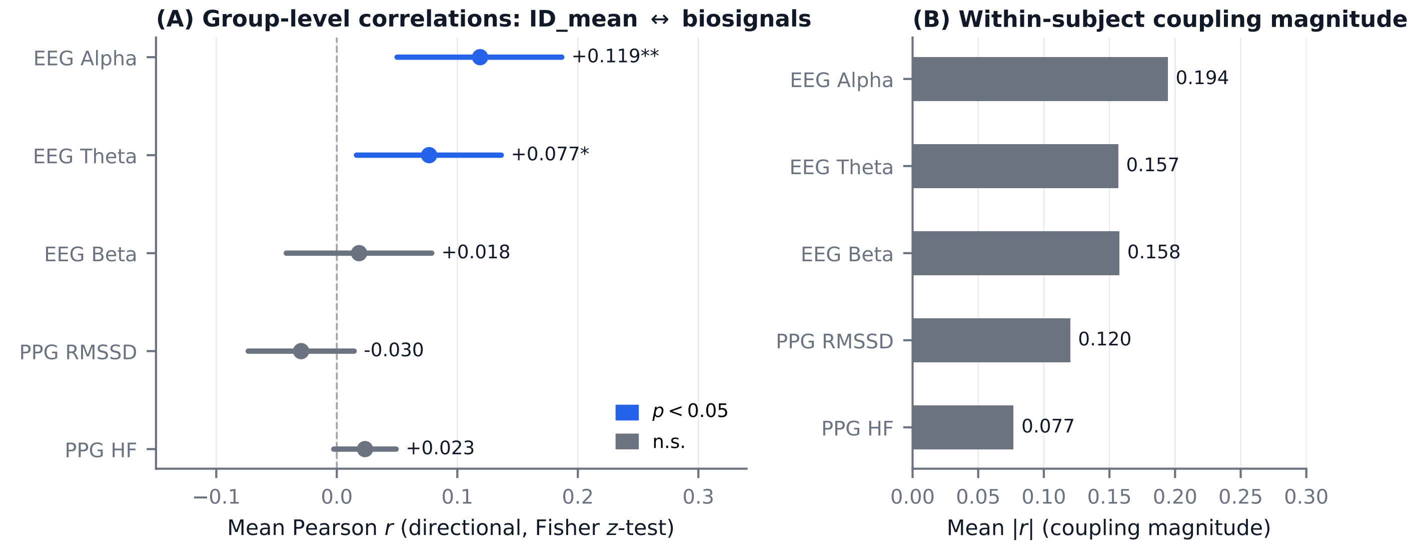

(A) Group-level correlations: ID_mean ↔ biosignals
EEG Alpha
EEG Theta
EEG Beta
PPG RMSSD
PPG HF
+0.119**
+0.077*
+0.018
-0.030
+0.023
p < 0.05
n.s.
−0.1
0.0
0.1
0.2
0.3
Mean Pearson r (directional, Fisher z-test)
(B) Within-subject coupling magnitude
EEG Alpha
EEG Theta
EEG Beta
PPG RMSSD
PPG HF
0.194
0.157
0.158
0.120
0.077
0.00
0.05
0.10
0.15
0.20
0.25
0.30
Mean |r| (coupling magnitude)
