# Supplementary material for: Tablet-Camera Iris Landmarks and EEG/PPG-Derived Arousal Indices During Naturalistic Reading: Geometric Confounding and Exploratory Within-Subject Associations
Source: Sensors (Basel). 2026 Oct 14;26(20):6505. doi: 10.3390/s26206505 (PMC13709060; doi:10.3390/s26206505)
Supplement: Supplementary file 1 [file sensors-26-06505-s001.zip › figures/fig3_r_distribution.pdf]

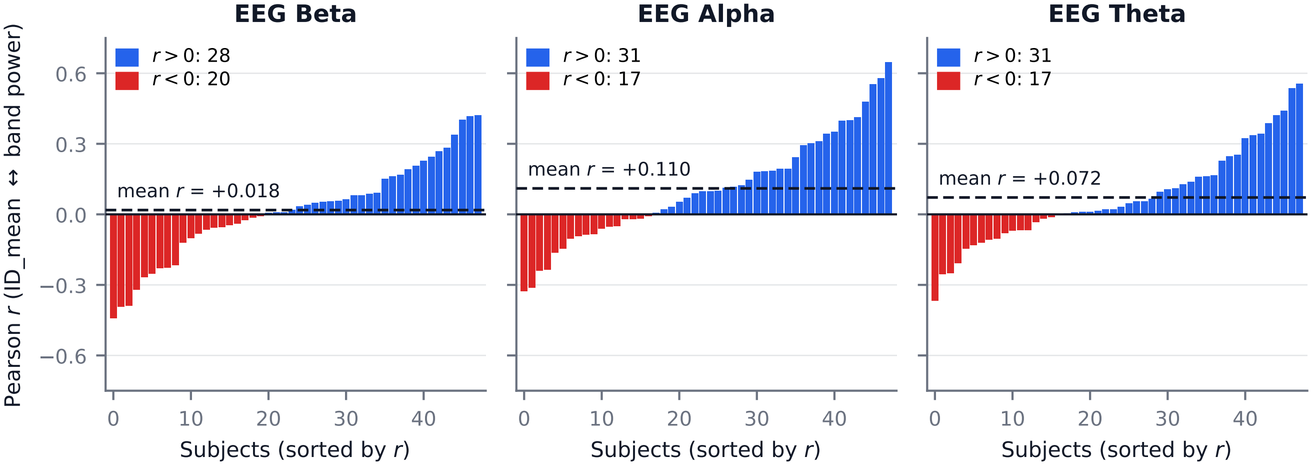

EEG Beta
EEG Alpha
EEG Theta
r > 0: 28
r < 0: 20
r > 0: 31
r < 0: 17
r > 0: 31
r < 0: 17
mean r = +0.018
mean r = +0.110
mean r = +0.072
Pearson r (ID_mean ↔ band power)
0.6
0.3
0.0
−0.3
−0.6
0
10
20
30
40
Subjects (sorted by r)
