# Supplementary material for: Tablet-Camera Iris Landmarks and EEG/PPG-Derived Arousal Indices During Naturalistic Reading: Geometric Confounding and Exploratory Within-Subject Associations
Source: Sensors (Basel). 2026 Oct 14;26(20):6505. doi: 10.3390/s26206505 (PMC13709060; doi:10.3390/s26206505)
Supplement: Supplementary file 1 [file sensors-26-06505-s001.zip › figures/fig5_facial_heatmap.pdf]

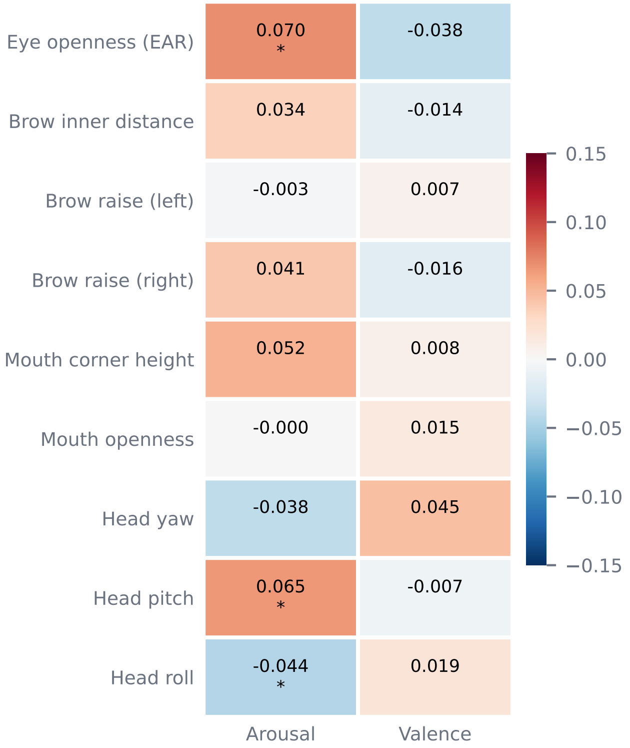
Eye openness (EAR)
Brow inner distance
Brow raise (left)
Brow raise (right)
Mouth corner height
Mouth openness
Head yaw
Head pitch
Head roll
Arousal
Valence
0.070 *
-0.038
0.034
-0.014
-0.003
0.007
0.041
-0.016
0.052
0.008
-0.000
0.015
-0.038
0.045
0.065 *
-0.007
-0.044 *
0.019
0.15
0.10
0.05
0.00
−0.05
−0.10
−0.15
Mean Pearson r (group-level)
