# Supplementary material for: Tablet-Camera Iris Landmarks and EEG/PPG-Derived Arousal Indices During Naturalistic Reading: Geometric Confounding and Exploratory Within-Subject Associations
Source: Sensors (Basel). 2026 Oct 14;26(20):6505. doi: 10.3390/s26206505 (PMC13709060; doi:10.3390/s26206505)
Supplement: Supplementary file 1 [file sensors-26-06505-s001.zip › figures/fig7_arousal_tracking.pdf]

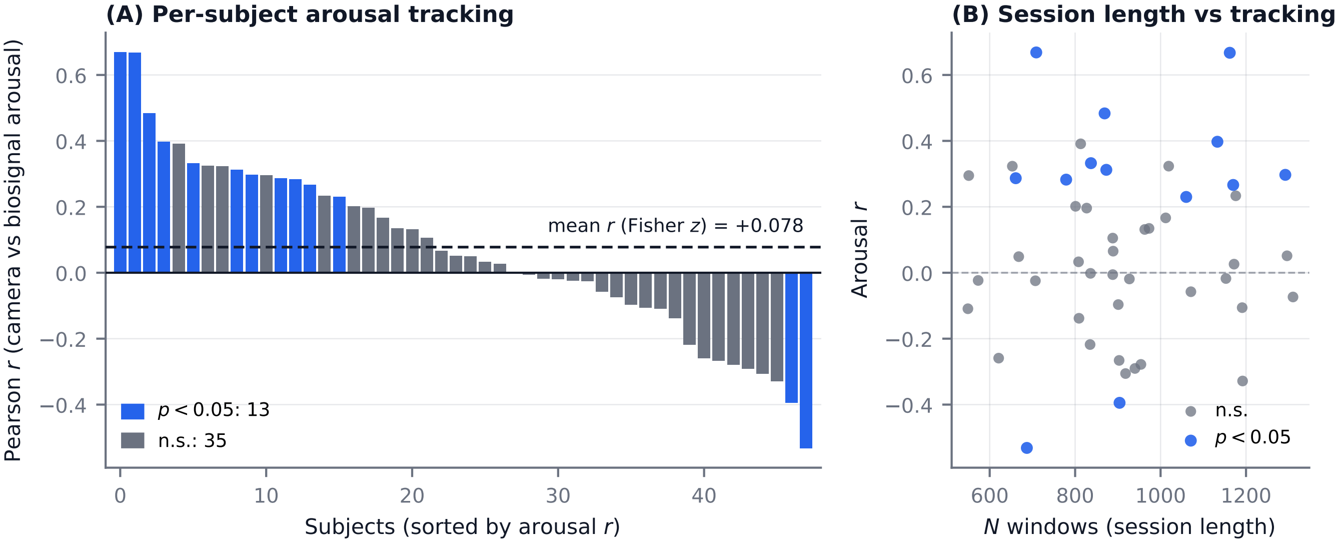

(A) Per-subject arousal tracking
Pearson r (camera vs biosignal arousal)
0.6
0.4
0.2
0.0
−0.2
−0.4
mean r (Fisher z) = +0.078
p < 0.05: 13
n.s.: 35
0
10
20
30
40
Subjects (sorted by arousal r)
(B) Session length vs tracking
Arousal r
0.6
0.4
0.2
0.0
−0.2
−0.4
n.s.
p < 0.05
600
800
1000
1200
N windows (session length)
