# Supplementary material for: Tablet-Camera Iris Landmarks and EEG/PPG-Derived Arousal Indices During Naturalistic Reading: Geometric Confounding and Exploratory Within-Subject Associations
Source: Sensors (Basel). 2026 Oct 14;26(20):6505. doi: 10.3390/s26206505 (PMC13709060; doi:10.3390/s26206505)
Supplement: Supplementary file 1 [file sensors-26-06505-s001.zip › figures/fig8_loso_cv.pdf]

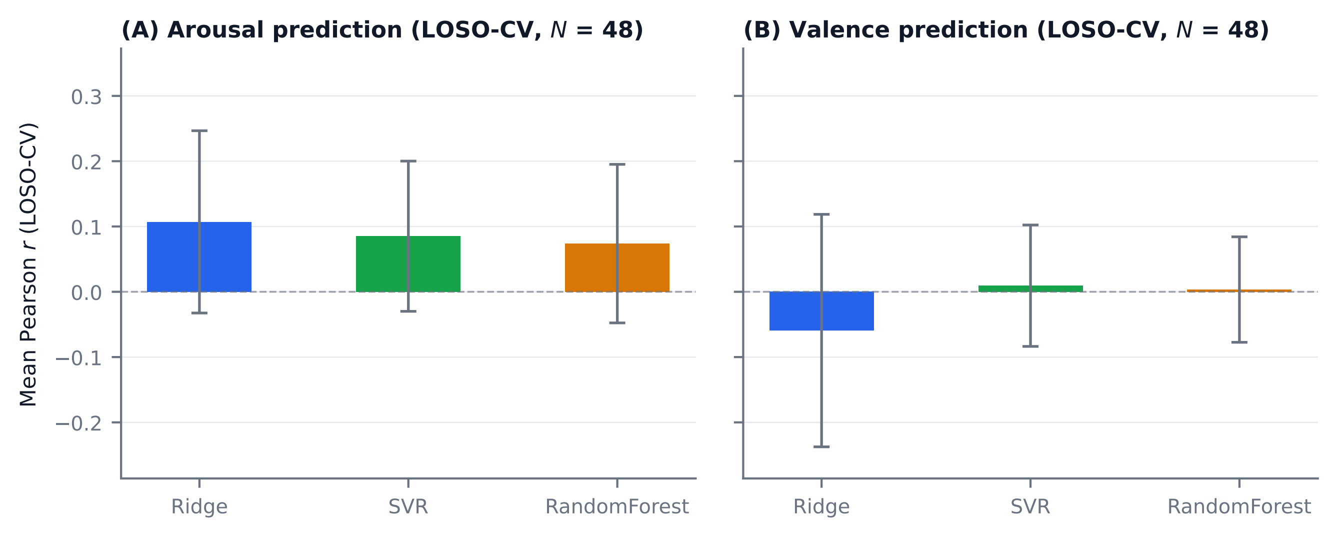
(A) Arousal prediction (LOSO-CV, N = 48)
(B) Valence prediction (LOSO-CV, N = 48)
Mean Pearson r (LOSO-CV)
0.3
0.2
0.1
0.0
−0.1
−0.2
Ridge
SVR
RandomForest
Ridge
SVR
RandomForest
