# Supplementary material for: Tablet-Camera Iris Landmarks and EEG/PPG-Derived Arousal Indices During Naturalistic Reading: Geometric Confounding and Exploratory Within-Subject Associations
Source: Sensors (Basel). 2026 Oct 14;26(20):6505. doi: 10.3390/s26206505 (PMC13709060; doi:10.3390/s26206505)
Supplement: Supplementary file 1 [file sensors-26-06505-s001.zip › figures/figS_granger.pdf]

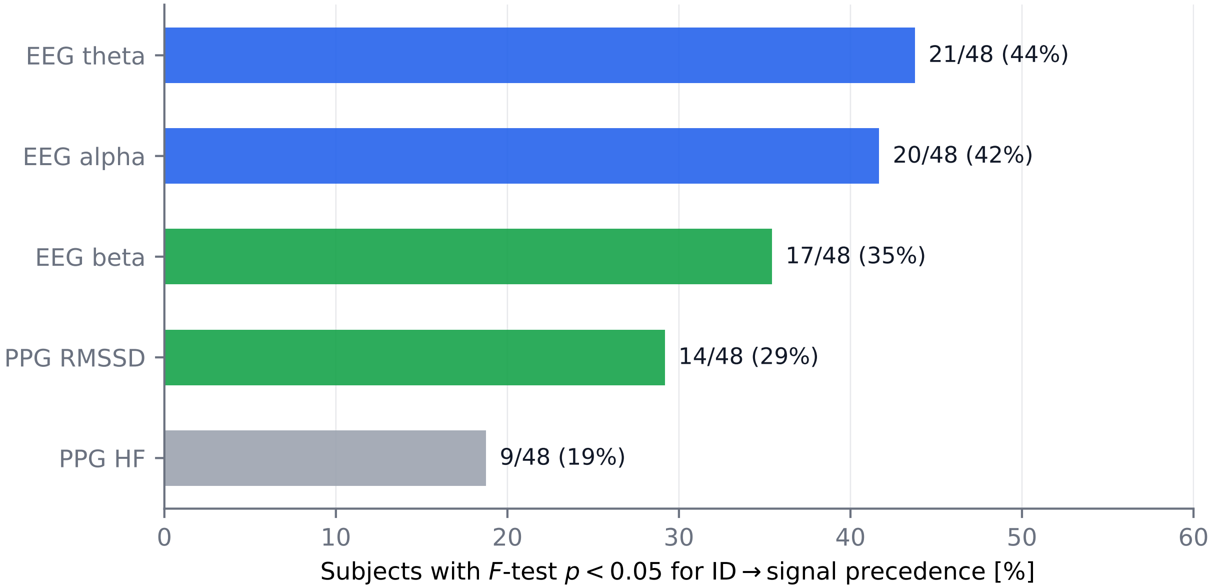

EEG theta
EEG alpha
EEG beta
PPG RMSSD
PPG HF
21/48 (44%)
20/48 (42%)
17/48 (35%)
14/48 (29%)
9/48 (19%)
0
10
20
30
40
50
60
Subjects with F-test p < 0.05 for ID → signal precedence [%]
