# Supplementary material for: Tablet-Camera Iris Landmarks and EEG/PPG-Derived Arousal Indices During Naturalistic Reading: Geometric Confounding and Exploratory Within-Subject Associations
Source: Sensors (Basel). 2026 Oct 14;26(20):6505. doi: 10.3390/s26206505 (PMC13709060; doi:10.3390/s26206505)
Supplement: Supplementary file 1 [file sensors-26-06505-s001.zip › figures/figS_pilot.pdf]

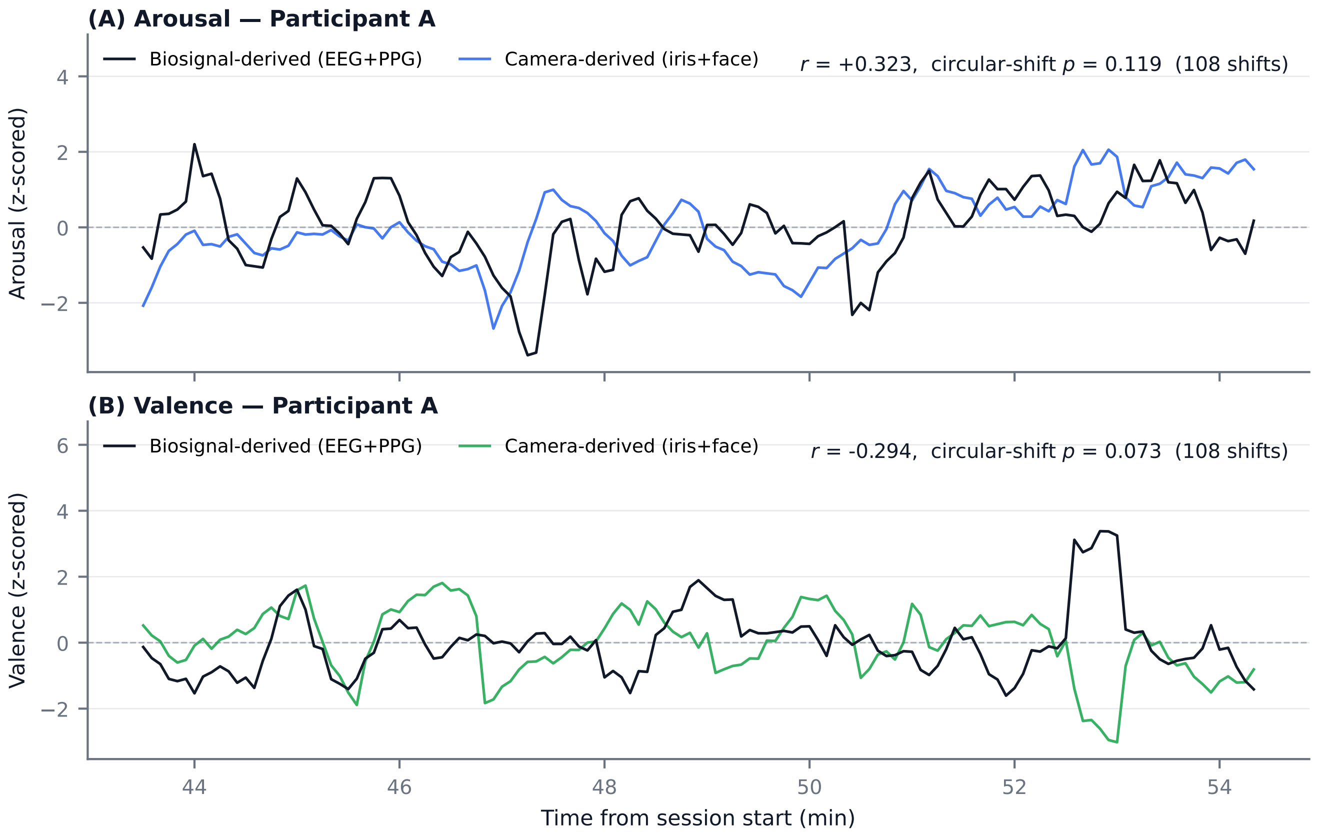

(A) Arousal — Participant A
Biosignal-derived (EEG+PPG)
Camera-derived (iris+face)
r = +0.323, circular-shift p = 0.119 (108 shifts)
Arousal (z-scored)
4
2
0
−2
(B) Valence — Participant A
Biosignal-derived (EEG+PPG)
Camera-derived (iris+face)
r = -0.294, circular-shift p = 0.073 (108 shifts)
Valence (z-scored)
6
4
2
0
−2
44
46
48
50
52
54
Time from session start (min)
