# Supplementary material for: Tablet-Camera Iris Landmarks and EEG/PPG-Derived Arousal Indices During Naturalistic Reading: Geometric Confounding and Exploratory Within-Subject Associations
Source: Sensors (Basel). 2026 Oct 14;26(20):6505. doi: 10.3390/s26206505 (PMC13709060; doi:10.3390/s26206505)
Supplement: Supplementary file 1 [file sensors-26-06505-s001.zip › figures/figS_ppg_sqi_soso.pdf]

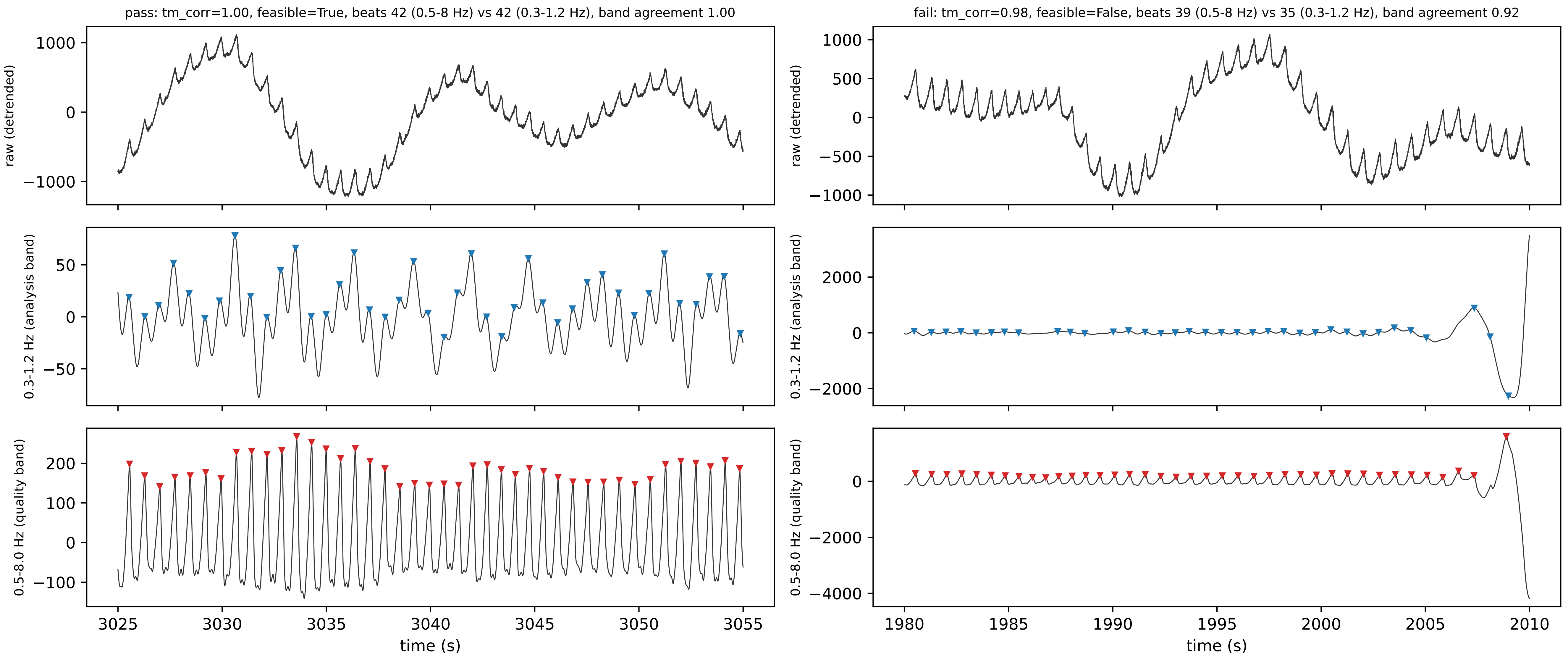

pass: tm_corr=1.00, feasible=True, beats 42 (0.5-8 Hz) vs 42 (0.3-1.2 Hz), band agreement 1.00
fail: tm_corr=0.98, feasible=False, beats 39 (0.5-8 Hz) vs 35 (0.3-1.2 Hz), band agreement 0.92
raw (detrended)
0.3-1.2 Hz (analysis band)
0.5-8.0 Hz (quality band)
time (s)
